# Small RNA Toxin‐Assisted Evolution of GC‐Preferred ErCas12a for Enhanced Genome Targeting Range

**DOI:** 10.1002/advs.202417105

**Published:** 2025-05-20

**Authors:** Zehua Chen, Junyuan Xue, Ziying Wang, Jinyuan Sun, Yinglu Cui, Tong Zhu, Huaiyi Yang, Ming Li, Bian Wu

**Affiliations:** ^1^ AIM center College of Life Sciences and Technology Beijing University of Chemical Technology Institute of Microbiology Chinese Academy of Sciences Beijing 100101 China; ^2^ State Key Laboratory of Microbial Diversity and Innovative Utilization Institute of Microbiology Chinese Academy of Sciences Beijing 100101 China; ^3^ Senior Department of Orthopedics the Fourth Medical Center of PLA General Hospital Beijing 100000 China; ^4^ University of Chinese Academy of Sciences Beijing 100049 China; ^5^ State Key Laboratory of Green Biomanufacturing Beijing 100029 China

**Keywords:** CRISPR/Cas12a, expanded PAM profiles, gene editing, RNA toxin‐assisted evolution

## Abstract

CRISPR/Cas12a, a promising gene editing technology, faces limitations due to its requirement for a thymine (T)‐rich protospacer adjacent motif (PAM). Despite the development of Cas12a variants with expanded PAM profiles, many genomic loci, especially those with guanine‐cytosine (GC)‐rich PAMs, have remained inaccessible. This study develops a small RNA toxin‐aided strategy to evolve ErCas12a for targeting GC‐rich PAMs, resulting in the creation of enhanced ErCas12a (enErCas12a). EnErCas12a demonstrates the ability to recognize GC‐rich PAMs and target five times more PAM sequences than the wild‐type ErCas12a. Furthermore, enErCas12a achieves efficient gene editing in both bacterial and mammalian cells at various sites with non‐canonical PAMs, including GC‐rich PAMs such as GCCC, CGCC, and GGCC, which are inaccessible to previous Cas12a variants. Moreover, enErCas12a effectively targets PAM sequences with a GC content exceeding 75% in mammalian cells, providing a valuable alternative to the existing Cas12a toolkit. Importantly, enErCas12a maintains high specificity at targets with canonical PAMs, while also demonstrating enhanced specificity at targets with non‐canonical PAMs. Collectively, this work establishes enErCas12a as a promising tool for gene editing in both eukaryotes and prokaryotes.

## Graphical abstract

ErCas12a is engineered to target GC‐rich PAMs using a small RNA toxin‐aided positive screening system. The resulting variant, enErCas12a, exhibits an expanded PAM profile and facilitates efficient gene editing in both bacterial and mammalian cells, while preserving high targeting specificity for both canonical and non‐canonical PAM targets.

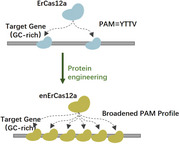

## 1 Introduction

The CRISPR/Cas system, originally discovered as the adaptive immune system in bacteria or archaea, has greatly impacted various fields such as life sciences, bioengineering, biomedicine, food, and agricultural sciences since its adaptation for gene editing.^[^ ^1^, ^2^, ^3^, ^4^ ^]^ A major limitation of this system is its strict reliance on a protospacer adjacent motif (PAM) sequence for target DNA recognition, which constrains various CRISPR‐based gene editing technologies, including base editing,^[^ ^5^, ^6^ ^]^ prime editing,^[^ ^7^ ^]^ or site‐specific DNA integration.^[^ ^8^, ^9^ ^]^ While PAM diversity can be extended through exploring natural CRISPR nucleases, their effectiveness are often hampered by the varying distribution of AT‐rich and GC‐rich DNA sequences across different genomic regions among species.^[^ ^10^, ^11^, ^12^ ^]^ Efforts to address this limitation have led to the development of CRISPR nucleases with expanded PAM compatibilities. Cas9, the most extensively studied CRISPR nuclease with a preference for G‐rich PAMs, has been engineered to develop variants capable of recognizing more AT‐rich PAMs, such as SpCas9‐NG,^[^ ^13^ ^]^ xCas9,^[^ ^14^ ^]^ SpRY,^[^ ^15^ ^]^ SaCas9‐KKH,^[^ ^16^ ^]^ and Sha2Cas9.^[^ ^17^ ^]^ However, such modifications often come at the cost of increased off‐target effects.^[^ ^18^ ^]^ In comparison, the Cas12a nuclease present reduced off‐target effects due to divergent cleavage mechanisms.^[^ ^19^, ^20^ ^]^ Ultrastable binding of Cas12a requires the same extent of sequence match (17‐bp PAM‐proximal matches) as target cleavage, while Cas9 requires only 9 bp and 16 bp PAM‐proximal matches for binding and cleavage, respectively.^[^ ^21^, ^22^, ^23^ ^]^ Additionally, Cas12a offers distinct features currently unavailable to Cas9. For instance, Cas12a has been shown to efficiently target spacer sequences with T‐rich PAMs. Interestingly, unlike Cas9, Cas12a can proceed with the cleavage process regardless of the presence of a PAM if the target sequence is already unwound.^[^ ^24^ ^]^ Moreover, Cas12a generates a 5′ overhang during DNA cleavage instead of the blunt ends left by Cas9 cleavage, providing advantages in homologous recombination‐mediated gene editing.^[^ ^25^ ^]^ Cas12a also processes its own CRISPR RNA (crRNA) from transcribed CRISPR arrays without additional accessory factors and triggers collateral cleavage of single‐stranded DNA upon target recognition, distinguishing it from Cas9.^[^ ^26^, ^27^ ^]^ Therefore, Cas12a has the potential to be an attractive alternative to exiting Cas9 variants. However, the widespread application of Cas12a has been limited by the requirement for T‐rich PAMs. Analysis of the human genome has revealed that GC‐rich regions are prevalent in coding sequences and other functional regulatory elements like histone methylation, with a higher potential for interfering with human complex traits and immunological diseases.^[^ ^28^, ^29^, ^30^ ^]^ Recent advancements in structure‐guided engineering have facilitated the development of Cas12a variants such as RVR,^[^ ^31^ ^]^RR,^[^ ^31^ ^]^ enAsCas12a^[^ ^32^ ^]^ and impLbCas12a,^[^ ^33^ ^]^ which exhibit reduced biases towards thymine (T) in PAM recognition and can recognize TC‐rich PAM sequences. In addition to broaden PAM compatibility, research efforts have focused on enhancing the editing efficiency of Cas12a, thereby broadening its applicability in human genomic manipulation.^[^ ^34^ ^]^ Despite these notable advancements, there are still challenges in accessing GC‐rich PAM sequences for Cas12a and its variants, which rise significant demands to broaden Cas12a's PAM repertoire, particularly within GC‐rich regions, to enhance its applicability and flexibility in manipulating and investigating the human genome.

In the subfamily of Cas12a, the *Eubacterium rectale*‐derived Cas12a nuclease, ErCas12a, has emerged as a promising tool due to its relatively relaxed PAM sequence and unrestricted availability, in addition to its proven effectiveness across various organisms including *Escherichia coli*, yeast, mammalian cells and plants.^[^ ^35^, ^36^, ^37^ ^]^ Improving ErCas12a's PAM compatibility, especially towards GC‐rich PAM sequences, could enhance the flexibility and effectiveness of the CRISPR/Cas12a platform in manipulating the human genome. In this study, we developed a highly sensitive positive selection system based on Cascade‐repressed Toxin (CreT), a class of small RNA toxins recently discovered,^[^ ^38^ ^]^ to facilitate the evolution of ErCas12a for the recognition of GC‐rich PAMs. The resulting mutant enErCas12a (K169R/D529R/K535N/D547N/K563R) exhibited heightened editing activity at target sites with non‐canonical PAMs including GC‐rich PAMs while maintaining robust activity on canonical PAMs in *E. coli* genome. Furthermore, we demonstrated that enErCas12a could serve as an effective genome‐editing tool with high specificity in human cells.

## 2 Results

### 2.1 Construction of the Positive Screening System Based on CreT RNA

**Figure 1:**
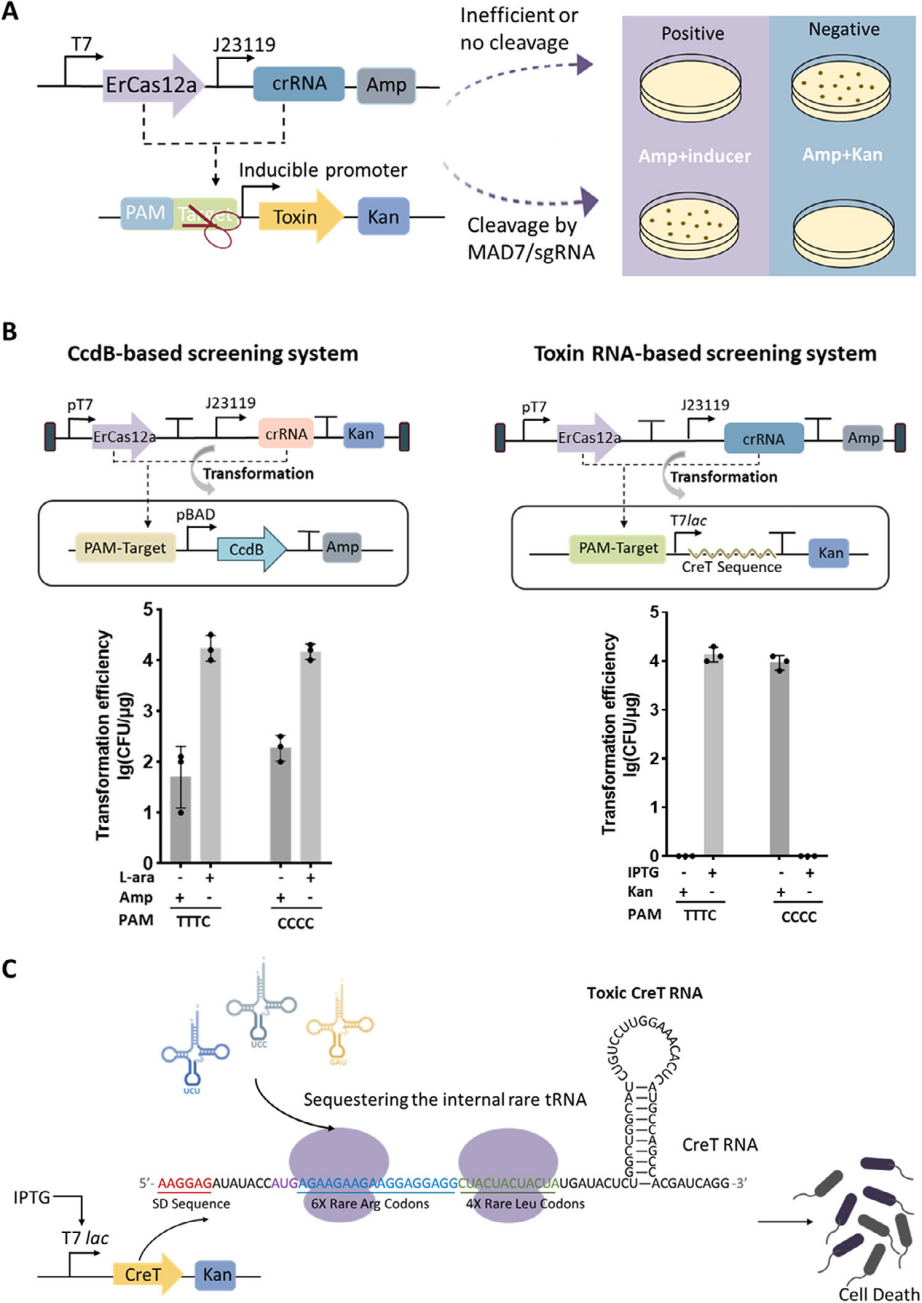
Toxin‐based screening system constructed in *E. coli*. A) Schematic of the toxin‐based positive selection assay and negative verification assay. B) Comparison of CcdB‐assisted screening system and toxic RNA‐aided screening system. The ErCas12a/crRNA expression plasmid, which was designed to target sites with PAM CCCC or PAM TTTC, was transformed into *E. coli* BW25113(DE3) containing either the CreT expression plasmid or the CcdB expression plasmid (p11‐LacY‐wtx1). Subsequently, an equal number of cells were spread onto agar plates supplemented with and without inducers. Data are mean ± s. d. of *n* =  3 independent experiments. C) Schematic of the CreT RNA sequence and the mechanism by which it caused bacterial death. The detail sequence of the toxic CreT RNA was shown.

A high‐throughput screening method is pivotal for the directed evolution of CRISPR nucleases to alter their PAM specificity. The introduction of a toxin gene links enzymatic DNA cleavage with the survival of host bacterial cells, demonstrating an effective and sensitive screening approach.^[^ ^39^ ^]^ Our toxin‐based screening system designed for the evolution of ErCas12a involved both a positive screening system and a negative verification system (**Figure** **1**A). In the positive screening system, toxin expression was eliminated when the ErCas12a/crRNA complex recognized and cleaved the target sequence on the reporter plasmid, promoting cell survival. On the contrary, in the negative system, cleavage of the reporter plasmid by the ErCas12a/crRNA complex led to the loss of kanamycin resistance, ultimately causing cell death in the presence of the kanamycin.

Kleinstiver et al. employed a positive screening system, with the assistance of CcdB toxic protein, to alter the PAM preference of spCas9.^[^ ^40^ ^]^ However, our attempts to evolve ErCas12a through the CcdB‐mediated screening system were unsuccessful, as the CcdB toxin was ineffective in causing cell death, for unknown reasons (Figure 1B; Figure S1, Supporting Information). In this study, we attempted to employ a small RNA toxin known as CreT (Cascade‐regulated toxin)^[^ ^38^ ^]^ to improve the positive screening platform in *E. coli*. The CreT RNA was found to sequester rare arginine tRNA^UCU^ in *Haloarcula hispanica* through the tandem AGA codons, thus inhibiting synthesis of crucial proteins and causing cell death.^[^ ^38^ ^]^ Analysis of codon usage in *E. coli* revealed AGG, AGA, CUA, AUA, CGA and CCC as the rarest codons, crucial for regulating endogenous protein expression.^[^ ^41^ ^]^ Exogenous rare codons, particularly tandem rare codons, have been shown to exhaust the endogenous tRNA of *E. coli* cells, resulting in reduced protein expression.^[^ ^42^ ^]^ To employ the CreT RNA as a lethal component in our screening system, we evaluated its toxicity under different promoters and determined that the isopropyl β‐D‐thiogalactopyranoside (IPTG)‐inducible T7*lac* promoter was the most effective in inducing toxicity (Figure S2A, Supporting Information). Furthermore, we increased the number of consecutive rare AGA, AGG codons, while also introducing additional consecutive rare CTA codons, leading to an almost 100% bacterial kill rate in our screening system (Figure S2B, Supporting Information). It was indicated that the archaeal RNA toxin CreT could be readily reprogrammed to produce cytotoxicity in bacterial cells, highlighting the programmability of such small RNA toxins.

The CreT‐based positive screening system was ultimately designed with two plasmids. The reporter plasmid, based on pET28a, carried the *cret* sequence under the regulation of the inducible T7*lac* promoter. Upon IPTG induction, *cret* was transcribed into a toxic RNA with a stem‐loop structure containing continuous rare codons of AGA, AGG, and CTA, which could trigger cell death by hijacking the corresponding rare tRNA in the cell (Figure 1C). The second plasmid served as an expression vector for Cas protein and crRNA, driven by the T7 and J23119 promoter, respectively. Importantly, our results demonstrated that the CreT‐based screening system effectively distinguished the capability of ErCas12a/crRNA to cleave targets with distinct PAMs (TTTC and CCCC), providing an efficient screening platform for subsequent evolution of ErCas12a (Figure 1B).

### 2.2 Evolution of ErCas12a to Target GC‐Rich PAMs

**Figure 2:**
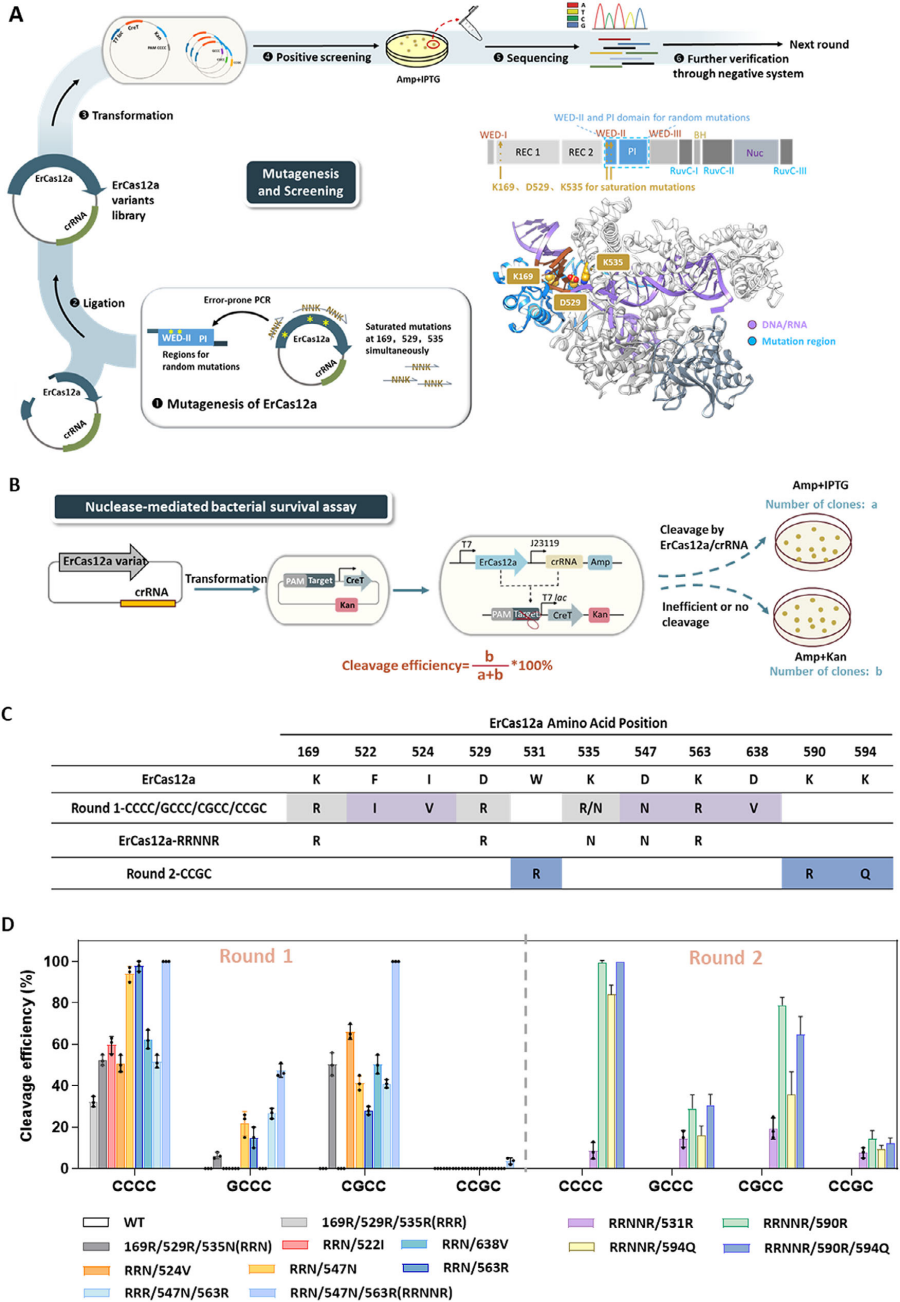
Evolution of ErCas12a to recognize GC‐rich PAMs. A) Schematic of the evolution of ErCas12a. Initially, a library of *ErCas12a* mutants was generated, incorporating saturated mutations at specific positions and random mutations within residues 513–677. These mutants were then introduced into *E. coli* cells containing CreT reporter plasmids with different PAM sequences, namely CCCC, GCCC, CGCC, and CCGC, and subjected to screening using the positive selection system. Surviving variants were subsequently sequenced to identify the mutations responsible for the altered PAM specificity. A new round of mutagenesis screening was carried out after further exploration of the obtained mutants. The structural model and detailed mutation regions of ErCas12a were shown in the right corner. Saturated mutation sites were marked as yellow spheres. Random mutation regions were marked as blue. PAM regions were marked as brown. B) Schematic of the nuclease‐mediated bacterial survival assay. The ErCas12a variant expression plasmid was transformed into *E. coli* cells containing the CreT reporter plasmid with different PAM sequences separately. After 1 h‐resuscitation, equal volumes of bacterial liquid were spread on agar plates following a 100‐fold dilution. Colonies counted on the IPTG/ampicillin plate labeled as (a), on kanamycin/ampicillin plate as b. The cleavage efficiency of each ErCas12a mutants was assessed by the ratio of (a) to (a+b). C) Summary of mutations obtained from the two rounds of evolutionary screening. In the first round for CCCC, GCCC, CGCC, and CCGC PAMs, mutations obtained from saturated mutagenesis were highlighted in gray, while those derived from random mutagenesis are indicated in purple. The mutant RRNNR serves as the starting point for the second round of mutagenesis. In the second round for CCGC PAM, newly acquired mutations were marked in dark blue. D) Combinatorial assembly and testing of mutations obtained from the two rounds of evolutionary screening for ErCas12a variants capable of cleaving target site with GC‐rich PAMs. The cleavage efficiency was determined as described above. Data are mean ± s. d. of *n* =  3 independent experiments.

With the CreT‐based screening system, we proceeded to enhance ErCas12a's ability to recognize GC‐rich PAMs. We initially introduced saturated mutations at positions 169, 529, and 539, known to influence PAM preference based on a previous studies,^[^ ^31^, ^32^, ^36^ ^]^ and then randomly mutagenized residues 513–677 within the WED‐II and the PI domain regions to generate a library of ErCas12a variants (**Figure** **2**A). To broaden ErCas12a's PAM recognition to include GC‐rich sequences, the mutagenesis library was transformed into *E. coli* cells harboring CreT reporter plasmids with four GC‐rich PAM sequences CCCC, GCCC, CGCC, and CCGC, which were typically challenging for commonly used Cas12a variants (Figure 2A). After transformation, surviving variants under selective conditions were isolated and sequenced to identify the mutations responsible for the altered PAM specificity. Following this initial screening and characterization of the promising mutations, a second round of mutagenesis was undertaken to further refine and enhance the PAM recognition capabilities of the identified variants by introducing additional mutations. This iterative approach led to the generation of ErCas12a variants with improved functionality towards GC‐rich PAMs. For the first round of screening, protein sequences of mutant regions from the surviving bacteria in the screening process were analyzed, and the most frequent substitutions were identified as K169R, F522I, I524V, D529R, K535R, K535N, D547N, K563R, and D638V (Figure S3, Supporting Information). In principle, bacterial cells with the CreT plasmid susceptible to ErCas12a cleavage could form colonies on the plate containing IPTG and ampicillin (the colony number labeled as a), while those with the CreT plasmid inaccessible to ErCas12a cleavage could form colonies on the plate containing kanamycin and ampicillin (the colony number labeled as b). We thus calculated the ratio of (a) to (a+b) to represent the cleavage efficiency of each ErCas12a derivate against a plasmid with a given PAM (Figure 2B). Following this, we conducted a preliminary assessment of the cleavage efficiency of different combinations of mutations targeting GC‐rich PAM sequences. Our results revealed that the ErCas12a‐RRNNR variant (K169R/D529R/K535N/D547N/K563R) exhibited improved efficiencies in targeting PAMs CCCC, GCCC, and CGCC, particularly achieving 100% efficiency in PAM CCCC and CGCC (Figure 2C,D). The amino acid mutations at positions 169 and 529 to arginine (R) were consistent with previous reports that extended the recognition of C‐rich PAM sequences.^[^ ^32^ ^]^ Additionally, our findings revealed that the mutation at position 535 to asparagine (N) significantly improved ErCas12a's ability to recognize PAM CGCC and GCCC, outperforming the effect seen with the arginine (R) mutation (Figure 2D). Furthermore, the introduction of mutations at positions 547 (to N) and 563 (to R) enhanced this recognition and binding capability (Figure 2D). Then the ErCas12a‐RRNNR variant was selected for further evolution. Subsequent efforts focused on introducing additional mutations to facilitate recognition of PAM CCGC, leading to the identification of three mutations W531R, K590R, and K594Q for further investigation (Figure 2C). While the addition of these single mutations improved recognition of PAM CCGC, they reduced the cleavage efficiency of the ErCas12a‐RRNNR variant in the presence of other GC‐rich PAMs, particularly the mutation W531R (Figure 2D). Consequently, the ErCas12a‐RRNNR variant, the ErCas12a‐RRNNRR variant (RRNNR/590R) and the ErCas12a‐RRNNRRQ variant (RRNNR/590R/594Q) were selected for further characterization.

### 2.3 Plasmids Depletion Assay Demonstrated Broadened PAM Profile of Obtained Mutants

**Figure 3:**
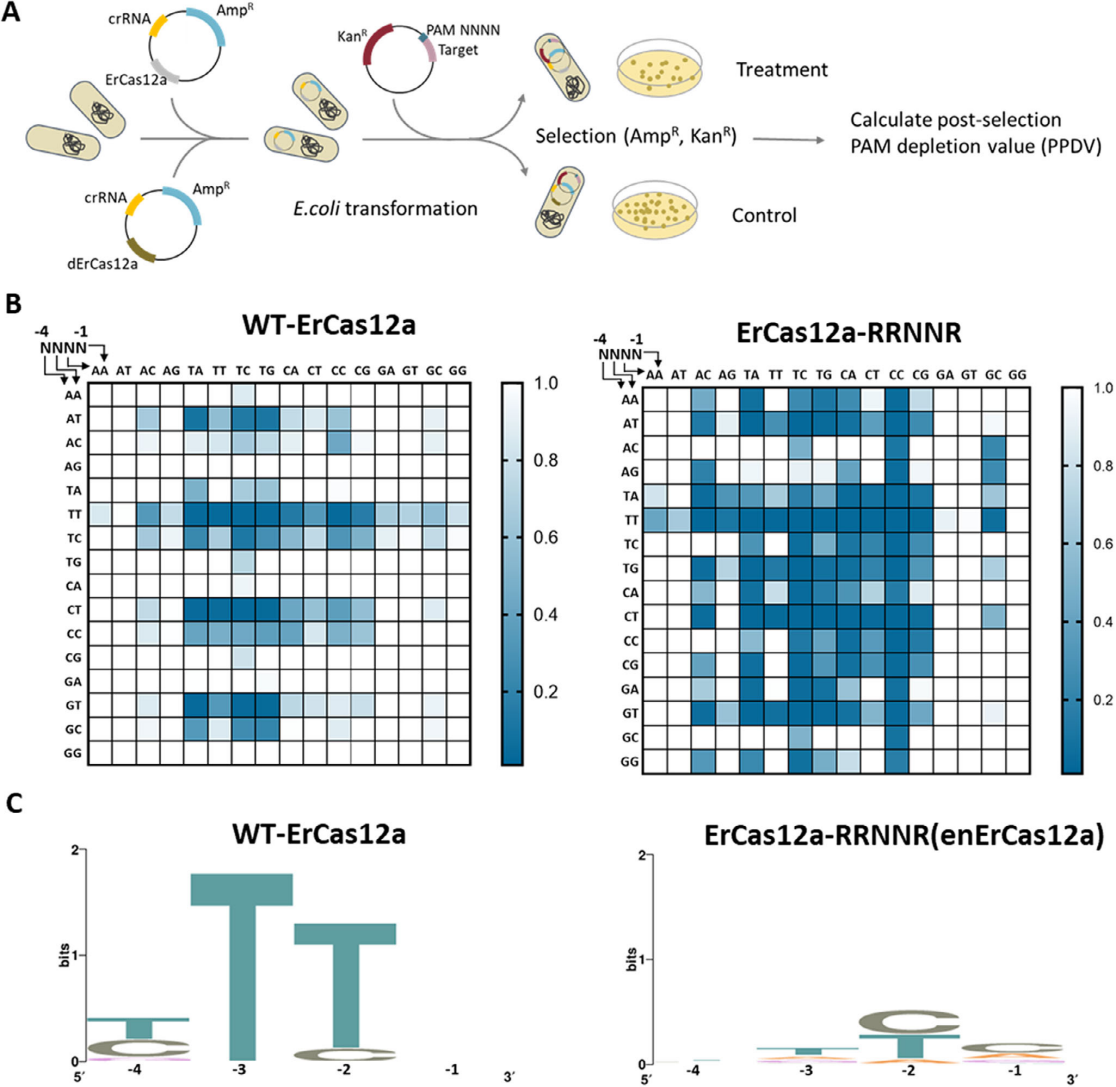
PAM specificities of wild‐type (WT) and the ErCas12a‐RRNNR variant. A) Schematic of the plasmid depletion assay. In the plasmid depletion assay, a library of plasmids bearing NNNN bases adjacent to a protospacer is tested for cleavage by ErCas12a in *E. coli*. Plasmids with PAM sequences refractory to ErCas12a enable cell survival due to the presence of a kanamycin‐resistance gene, whereas plasmids harboring targetable PAMs are depleted from the library. Sequencing the remaining (uncleaved) population of plasmids allows for the calculation of the post‐selection PAM depletion value (PPDV), which serves as an estimate of ErCas12a activity against those PAMs by comparing the post‐selection frequency to the pre‐selection frequency. A catalytically dead ErCas12a (dErCas12a) was used as a control in the experiment. B) PAM preference profiles, assessed by mean PPDVs, for WT ErCas12a and the ErCas12a‐RRNNR variant. The mean PPDVs were calculated from four replicates, with two against each of two distinct spacer sequences. C) Web logos of the most depleted PAMs (with a mean of PPDVs smaller than 0.1) for WT and the ErCas12a‐RRNNR (enErCas12a) variant.

To comprehensively characterize the PAM specificities of the obtained mutants, we performed the plasmids depletion assay as previously described,^[^ ^40^ ^]^ which allowed us to acquire the PAM preference profiles by identifying the relative cleavage of DNA plasmids with randomized PAMs and quantified as a post‐selection PAM depletion value (PPDV) (**Figure** **3**A). The experiments were carried out using wild‐type ErCas12a and three ErCas12a mutants separately, utilizing libraries with two different spacer sequences, each containing four randomized bases (NNNN) or five randomized bases (NNNNN) in the location of PAM. With wild‐type ErCas12a, the most depleted PAMs, based on the mean of PPDVs obtained from the two libraries, were the YTTN (Y = C, T) PAMs as reported (Figure 3B,C; and Table S1, Supporting Information). ErCas12a also targeted other PAM sequences, including TTCV and RTTV (R = A, G), albeit at lower rates (Figure 3B; and Table S1, Supporting Information). By contrast, the ErCas12a‐RRNNR variant demonstrated that PAMs with mean PPDVs of less than 0.1 included not only the BTTN (B = C, T, G) PAMs but also ATTV, NNCC, YNTC, TNCA, and GATV PAMs (Figure 3B; and Table S1, Supporting Information). In particular, ErCas12a‐RRNNR was able to target 40 out of the 176 (22.7%) GC‐rich PAMs (two or more C/G within ‐1 to ‐4 positions of PAM sequence), significantly outperforming the wild‐type which recognized only 5 PAMs (2.8%). Further research revealed that additional mutations did not result in preferable mutants, although they exhibited enhanced tolerance to guanine (G) at ‐2 position of PAM sequences (Figure S4A,B and Table S1, Supporting Information). Furthermore, none of the variants exhibited a significant preference for the ‐5 position of the PAM sequence (Figure S4C and Table S1, Supporting Information). Thus, the ErCas12a‐RRNNR variant, showing enhanced PAM compatibility and an improved affinity for C at the ‐2 position within the PAM sequence, was referred to as enhanced ErCas12a (enErCas12a) (Figure 3C).

### 2.4 EnErCas12a Could Target Non‐Canonical PAMs and Achieve Efficient Gene Editing in Bacteria

**Figure 4:**
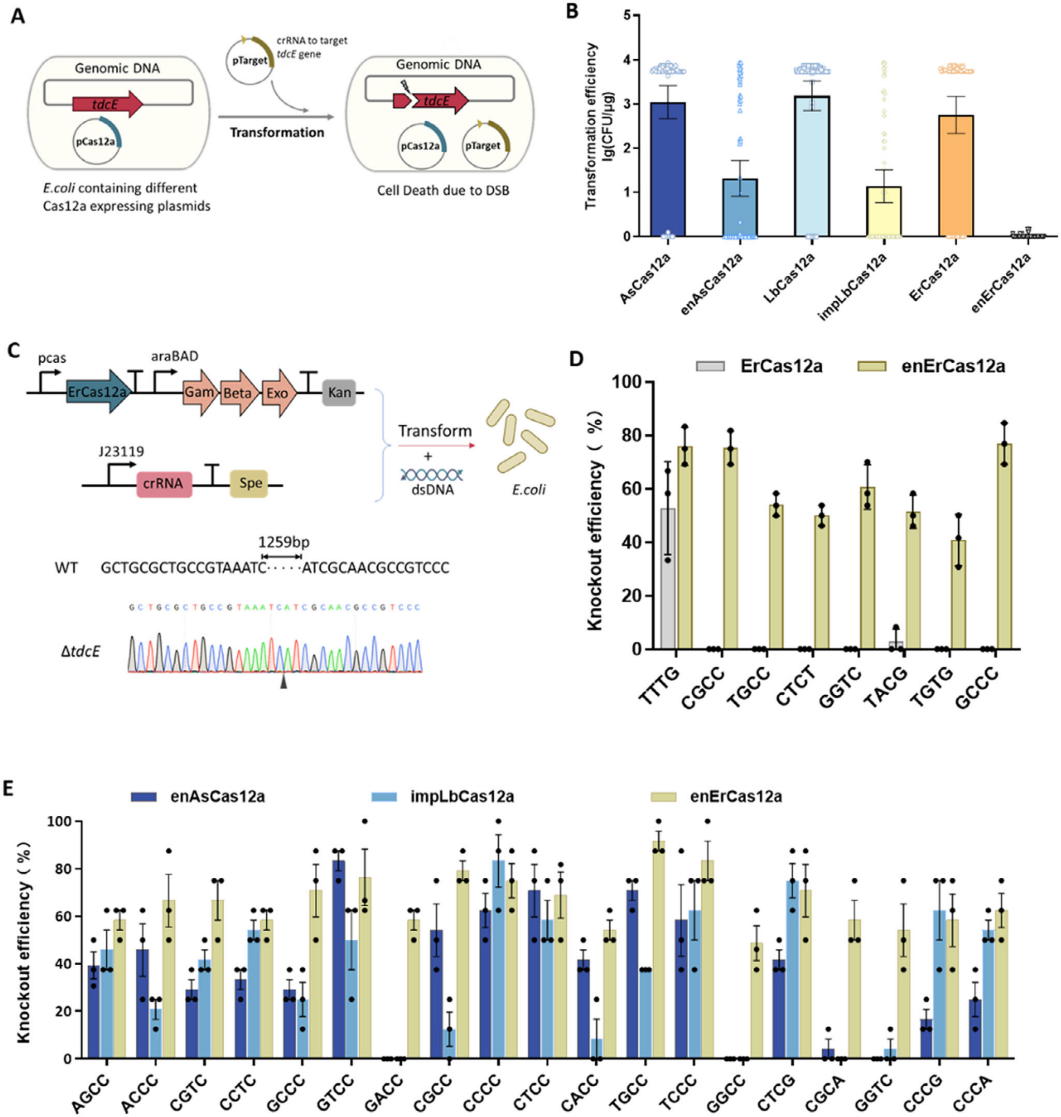
Genome editing in bacteria. A) Scheme of Cas12a‐mediated genome interference assay in *E. coli*. Different Cas12a nucleases were employed to induce DBS in the *tdcE* gene of the *E. coli* genome, which resulted in bacterial death. B) Summary of the transformation efficiency of six Cas12a nucleases, including AsCas12a, enAsCas12a, LbCas12a, impLbCas12a, ErCas12a, and enErCas12a across the 66 PAMs in the genome interference assay. Transformation efficiency was calculated by counting the colonies on plates and multiplying the value by the dilution ratio. Every assay has been conducted with three biological replicates. The average and standard deviation of transformation efficiency (colony‐forming unit per µg plasmid DNA, CFU/µg) were calculated based on log‐transformed data. C) Scheme of ErCas12a mediated genome editing in *E. coli*. The 1259‐bp fragment deletion of *tdcE* gene in *E. coli* genome, confirmed by sanger sequencing. D) Knockout efficiency of *tdcE* gene by WT and the enErCas12a variant at 8 target sites bearing different PAM sequences. Data are mean ± s. d. of *n* =  3 independent experiments. E. Knockout efficiency of *tdcE* gene by enAsCas12a, impLbCas12a and enErCas12a at 19 target sites bearing different GC‐rich PAM sequences. Data are mean ± s. d. of *n* =  3 independent experiments.

Based on the PAM preference profile of the enErCas12a variant, we investigated its potential for genome editing initially in bacteria. We programed the Cas12a nucleases to induce a (double strand breads) DSBs in the *tdcE* gene of *E. coli* genome, which in principle lead to cell death because the majority of bacteria is deficient in repairing DSBs due to the lack of the non‐homologous end joining (NHEJ) DNA repair pathway (**Figure** **4**A). By comparing transformation efficiency of such a self‐targeting plasmid, we assessed the targeting efficiencies of six Cas12a proteins, including AsCas12a, enAsCas12a, LbCas12a, impLbCas12a, ErCas12a, and enErCas12a across the 66 PAMs (mean PPDVs <0.1 for enErCas12a). Our findings revealed that WT ErCas12a was capable of targeting YTTN, RTTV, and TTCV PAMs while enErCas12a demonstrated broad targeting and cleavage capabilities across all 66 PAMs. Notably, both impLbCas12a and enAsCas12a exhibited reduced efficiency compared to enErCas12a when targeting GC‐rich PAMs, such as GGCC, CGTC, GACC, GGTC, GCCC, CGCA, GACC, CCCG (Figure 4B; Figure S5, Supporting Information). Subsequently, we introduced lambda‐red recombinases along with double‐stranded oligonucleotides serving as repair templates to facilitate homology‐directed repair (HDR) for gene knockout in *E. coli* (Figure 4C).^[^ ^43^ ^]^ The recombination donor DNA and crRNA‐expressing plasmids, each targeting unique sites with different PAMs, were separately introduced into *E. coli* containing different Cas12a expression plasmids. This resulted in the successful deletion of a 1259‐bp fragment within the *tdcE* gene of the *E. coli* genome (Figure 4C). Consistent with the PAM preference profile, we observed efficient gene editing for the enErCas12a variant at 8 target sites with diverse PAM sequences, achieving editing efficiencies ranging from 40% to 80%. In contrast, WT ErCas12a demonstrated effective editing only at the site with a TTTG PAM (Figure 4D; Figure S6A,B, Supporting Information). Further, we evaluated the gene knockout efficiencies of enAsCas12a, impLbCas12a, and enErCas12a across 19 target sites with high‐GC PAM sequences. The results demonstrated that enErCas12a efficiently achieved gene knockouts at all tested targets, demonstrating superior performance at PAMs GCCC, GACC, GGCC, CGCA, and GGTC compared to either enAsCas12a or impLbCas12a (Figure 4E). These data underscored the capability of the enErCas12a variant to effectively target and edit genome loci with non‐canonical PAMs in bacteria.

### 2.5 Genome Editing with enErCas12a in Human Cells

**Figure 5:**
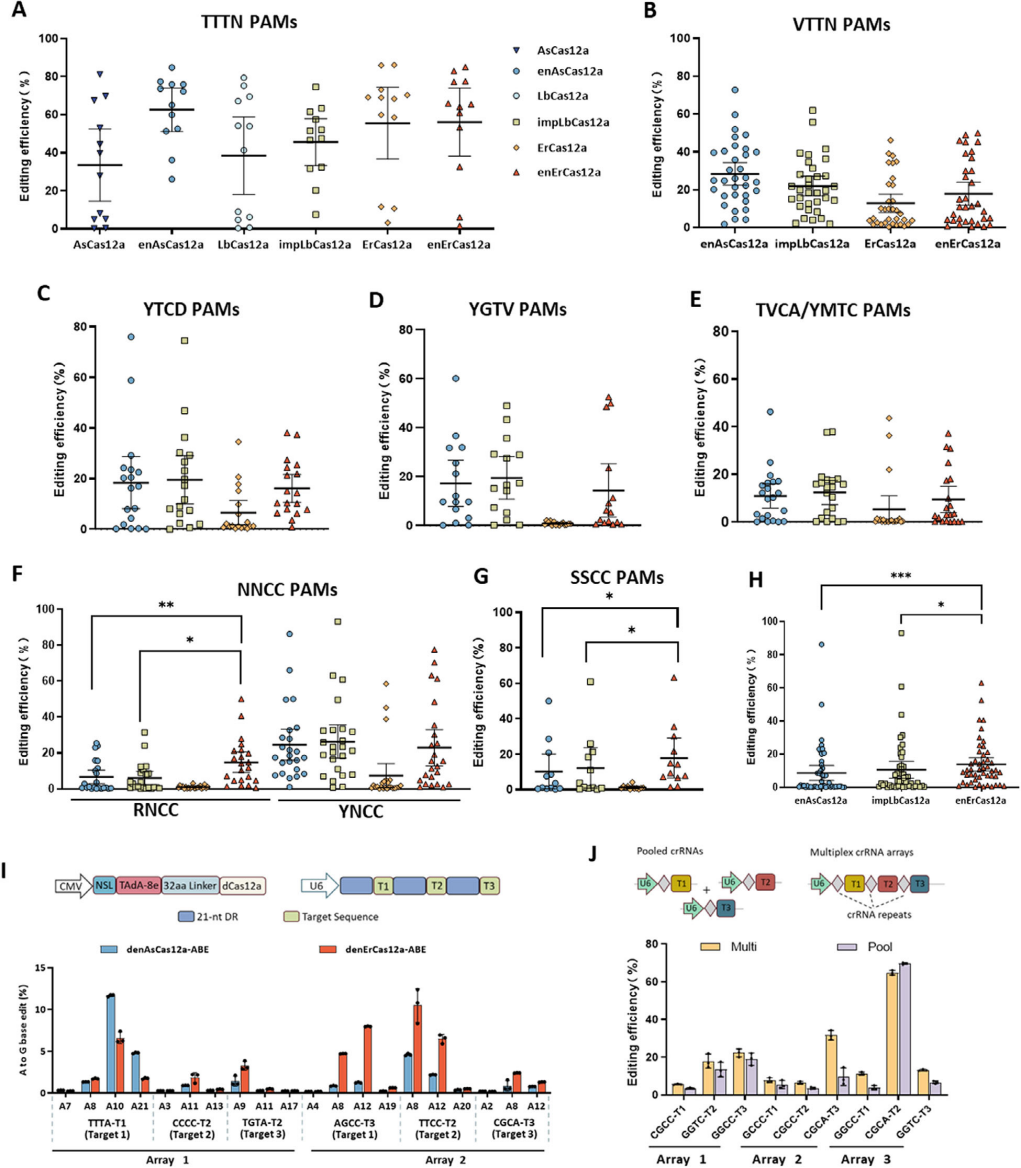
Genome editing with the enErCas12a variant at endogenous loci in HEK293T cells. A) Summary of the indel frequencies mediated by LbCas12a, AsCas12a, ErCas12a and their variants at 12 target sites with TTTN PAMs. Data are mean ± s. d. of *n* =  3 independent experiments. B–G) Summary of the editing efficiencies mediated by the enAsCas12a, impLbCas12a, ErCas12a and enErCas12a nucleases on targets with VTTN PAMs (B), YTCD PAMs (C), YGTV PAMs (D), TVCA/YMTC PAMs (E), NNCC PAMs (F), and SSCC PAMs (G). Data are mean ± s. d. of *n* =  3 independent experiments. H) Comparison of the activities of enAsCas12a, impLbCas12a and enErCas12a on targets with PAMs containing more than 75% GC content. Data are mean ± s. d. of *n* =  3 independent experiments. I) Comparison of the multiplex base editing efficiency of denAsCa12a‐ABE and denErCas12a‐ABE in HEK293 cells. A to G base editing efficiency of denAsCa12a‐ABE and denErCas12a‐ABE across six endogenous targets using two multiplex crRNA arrays, each designed to target 3 genomic loci containing different PAMs. The endogenous loci targeted by each array and the PAM sequences were shown. Data are mean ± s. d. of *n* =  3 independent. *, *p* < 0.05; **, *p* < 0.01; ***, *p* < 0.001. J) Assessment of editing efficiencies with enErCas12a when using pooled crRNA plasmids or multiplex crRNA arrays expressing three crRNAs targeted to genomic loci with GC‐rich PAMs. Data are mean ± s. d. of *n* =  3 independent experiments.

Motivated by effective genome editing in bacteria, we proceeded to evaluate the editing activity of enErCas12a in human cells. We previous identified 66 PAMs with average PPDVs of less than 0.1 from the PAM preference profile of enErCas12a. Then we selected 198 endogenous target sites containing these 66 diverse PAMs to perform genome editing in HEK293T cells (Table S2, Supporting Information). We compared the genome‐editing efficiencies of ErCas12a and the enErCas12a variant with those of AsCas12a, enAsCas12a, LbCas12a, and impLbCas12a at 12 target sites with the TTTN PAMs in HEK293T cells. AsCas12a, enAsCas12a, LbCas12a, impLbCas12a, ErCas12a, and enErCas12a generated indels at these 12 sites with 33.54%, 62.64%, 38.51%, 45.60%, 55.53%, and 56.13% frequencies on average, respectively (**Figure** **5**A). Furthermore, we extended our comparison to 186 target sites with non‐ canonical PAMs, assessing the genome‐editing efficiencies of ErCas12a and enErCas12a alongside enAsCas12a and impLbCas12a (Figure 5B–G; Figure S7, Supporting Information). The wild‐type ErCas12a exhibited editing capabilities for YTTV PAMs and selectively targeted a subset of RTTV PAMs, along with TCTC and TTCV PAMs (Figure S7, Supporting Information). In comparison, enErCas12a demonstrated similar editing activity for the canonical YTTN PAMs, while achieving enhanced editing efficiencies for targets with non‐canonical PAMs (Figure 5A–G). Comparative analyses demonstrated that enErCas12a exhibited superior editing efficiency on targets with RNCC and SSCC PAMs (Figure 5F,G), while it did not surpass the editing efficiency of enAsCas12a and impLbCas12a for targets featuring NTTN, YTCD, YGTV, TVCA, YMTC, and YNCC PAMs (Figure 5A–F). When considering PAM sequences with a GC content over 75%, the overall performance of enErCas12a was relatively higher (Figure 5H). The results indicated that enErCas12a possessed partially overlapping yet still different PAM preferences with impLbCas12a and enAsCas12a, especially for GC‐rich PAM, collectively extending the range of targets available for Cas12a nucleases. Similar results were obtained in Hela cells (Figure S8A–C, Supporting Information). Our findings validated the greatly extended PAM profile of enErCas12a within human cells, further broadening the range of DNA sequences amenable to targeting by the Cas12a nuclease.

To explain the effects of the identified mutations on PAM recognition, we used AlphaFold3 to predict the structure of enErCas12a (Figure S9A, Supporting Information). The D529R and K535N mutations, located in close proximity to the PAM sequence, are likely to have a direct impact on PAM recognition. Structural analysis indicates that the R529 mutation may form significantly stronger hydrogen bond interactions with the dC (‐3*) nucleotide compared to the wild‐type D529, potentially enhancing the binding affinity for G‐containing PAMs (Figure S9B, Supporting Information). Additionally, the N535 mutation appears to facilitate hydrogen bonding with the phosphate backbone of nucleotides that are complementary to the PAM, rather than engaging in base‐specific interactions, thereby contributing to relaxed PAM recognition (Figure S9C, Supporting Information). Apart from these two mutations, the remaining mutations occur at locations distant from the PAM sequence. These mutations may induce subtle structural alterations to enhance overall non‐specific binding to DNA.

It has been reported that the M537R and F870L mutations markedly enhances the activity of AsCas12a without compromising its high intrinsic specificity.^[^ ^34^ ^]^ Therefore, we introduced the analogous mutation (F840L) to enErCas12a, yielding the enErCas12a‐F840L variant, which exhibited increased activity (Figures S10 and S11, Supporting Information). However, this enhancement, particularly evident for non‐canonical PAMs, was accompanied by a significant increase in off‐target effects (Figure S12A,B, Supporting Information).

To leverage the unique capabilities of Cas12a in multiplexed genome editing, we utilized engineered enErCas12a‐mediated Adenine Base Editing (ABE) to achieve simultaneous editing at multiple genomic loci using a single CRISPR array and compared to denAsCas12a‐ABE.^[^ ^44^ ^]^ We tested in HEK293T cells with a single array containing three tandem crRNAs designed to target distinct loci featuring various PAMs. As expected, the A to G conversions observed were predominantly restricted to the editing window ranging from A8 to A13, consistent with previous reports described for Cas12a base editors (Figure 5I).^[^ ^44^ ^]^ Notably, denErCas12a‐ABE demonstrated enhanced editing activity at loci containing AGCC and TGCC PAMs, while denAsCas12a‐ABE exhibited superior editing efficiency on the target with TTTA PAM (Figure 5I). These findings highlight the potential of enErCas12a for multi‐target editing applications. We also designed multiplex arrays encoding three crRNAs that target to genomic loci with GC‐rich PAMs to generate small genomic deletions using enErCas12a. These crRNAs were expressed from either poly‐crRNA transcripts or pools of single crRNA plasmids, and we observed comparable or improved deletion efficiencies with the multiplex arrays (Figure 5J).

### 2.6 EnErCas12a Demonstrated a High Targeting Specificity When Tolerating Non‐Canonical PAMs

**Figure 6:**
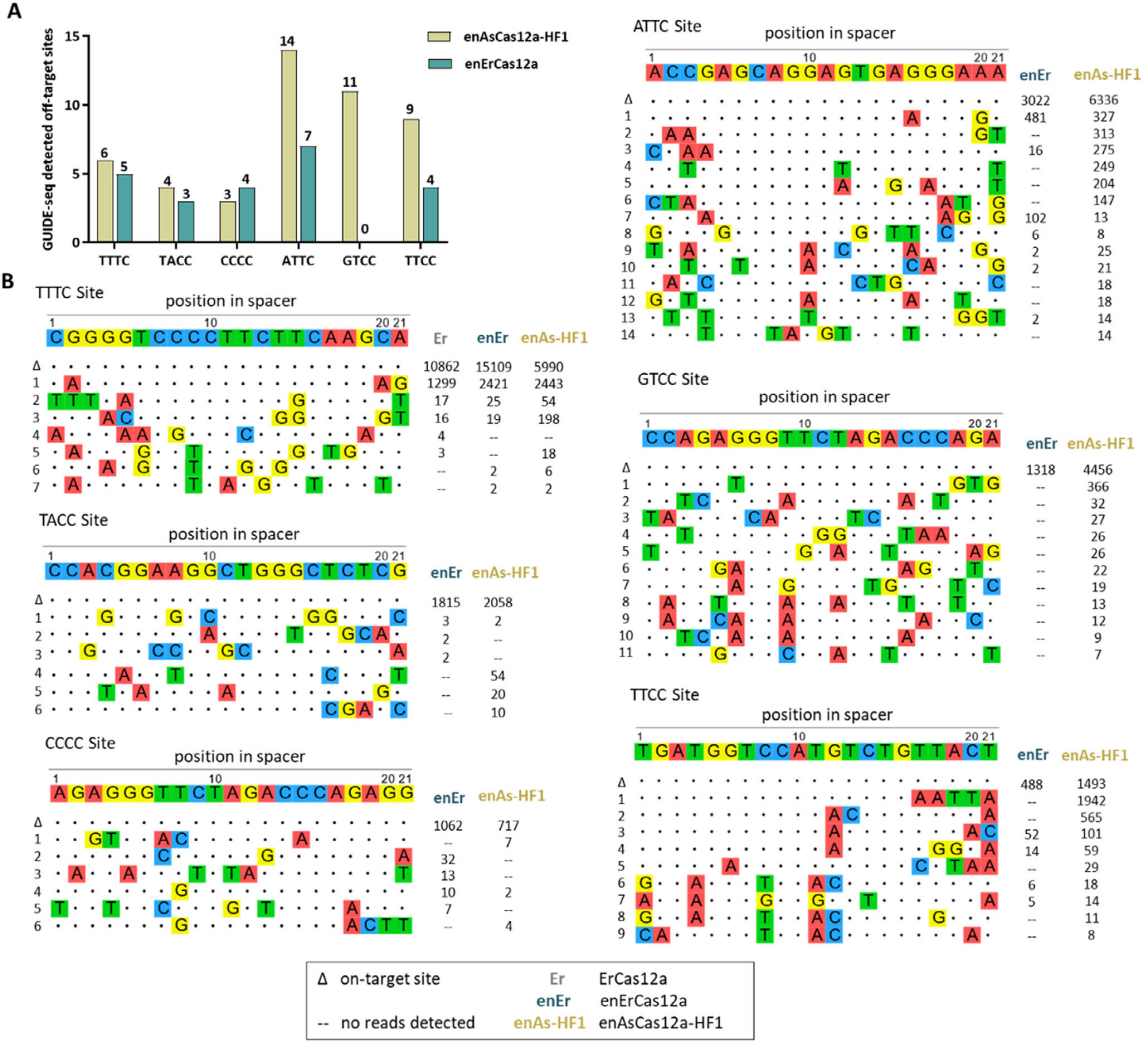
Characterization of enErCas12a specificity. A) Histograms illustrating the number of GUIDE‐seq detected off‐target sites for enErCas12a and enAsCas12a‐HF1 on sites with canonical TTTC PAM or non‐canonical PAMs. B) GUIDE‐seq genome‐wide specificity profiles for ErCas12a, enErCas12a, and enAsCas12a‐HF1 each paired with crRNAs targeting sites with TTTC PAM or non‐canonical PAMs. Mismatched positions in off‐target sites were highlighted in color and GUIDE‐seq read counts were shown to the right of the sequences.

Finally, we evaluated the mismatch tolerance of enErCas12a mediated gene editing by treating HEK293T cells with enErCas12a together with a corresponding crRNA containing one or two mismatches with the target site. For the target site with PAM TTTC, both the wild‐type ErCas12a and enErCas12a exhibited similar specificity, showing the ability to tolerate most single‐base mismatches while being intolerant to two‐base mismatches in the PAM proximal region (Figure S12A, Supporting Information). Interestingly, for the target site with PAM TACC and PAM GCCC, the enErCas12a variant displayed a high level of intolerance toward all single or double mismatches across the guide sequences (Figure S12B, Supporting Information). Furthermore, we assessed the genome‐wide specificity of enErCas12a in human cells using GUIDE‐seq, comparing its performance to that of the previously characterized enAsCas12a‐HF1 mutant, which exhibited high specificity and a broad PAM recognition range. Our experiments involved six crRNAs targeting sites with canonical TTTC PAM as well as various non‐canonical PAMs. The GUIDE‐seq results revealed fewer detectable off‐targets for enErCas12a compared to enAsCas12a‐HF1, particularly at sites with non‐canonical PAMs (**Figure** **6**A,B). Additional GUIDE‐seq experiments targeted to sites with GC‐rich PAMs (GCCC, CGCC, GGCC, GGTC) revealed that enErCas12a had few off‐target sites and low GUIDE‐seq read counts at these sites. (Figure S13, Supporting Information). Overall, enErCas12a demonstrated high editing specificity in human cells, particularly at sites with non‐canonical PAMs, suggesting its potential as a promising gene editing tool in gene therapy.

## 3 Discussion

In the directed evolution of CRISPR nucleases, researchers have employed toxic elements to establish a connection between Cas nucleases mediated DNA cleavage and cell survival, proving a highly efficient and sensitive screening approach. In this study, we utilized the small RNA toxin CreT, which sequesters the rare arginine tRNA and causes cytotoxicity, to construct a highly robust screening system for evolving the Cas12a nuclease (Figure 1). Notably, this screening system exhibited greater stringency compared to the traditional system that employs the protein toxin CcdB. This enhanced stringency can likely be attributed to the smaller and less complex genetic components of the small RNA toxin system, which can rapidly arrest translation after being transcribed. In contrast, CcdB synthesis relies on both bacterial transcription and translation processes and exerts its effects by inhibiting DNA gyrase, thereby causing double‐strand DNA breaks. In principle, bacteria have more chances to tolerate CcdB toxicity to some extent, e.g. by lowering the transcription/translation activity, enhancing DNA repair pathways, slowing down the DNA replication process, or through other mechanisms, which need be further investigated. It is worth mentioning that the RNA toxin CreT was originally discovered in *haloarchaea* ^[^ ^37^, ^45^ ^]^ and can be easily customized to halt the growth of *E. coli* cells by understanding the codon usage frequency of this bacterium, showcasing the flexibility and programmability of such RNA toxins. In addition, more RNA toxins (regulated by type I CRISPR‐Cas systems) with differing mechanisms have been recently uncovered in different bacteria and archaea, which offers promising opportunities for developing RNA toxin‐based screening systems in diverse microorganisms. This presents a valuable alternative to the limited scope of the CcdB‐based selection system and the phage‐assisted continuous evolution (PACE), predominantly used in *E. coli*.^[^ ^46^ ^]^ We anticipate the prospective application of our evolutionary approach in the engineering of nucleic acid‐associated functional elements such as transcription factor, endonuclease, exonuclease and CRISPR nuclease to meet the demands for specificity, efficiency and compatibility in various applications.

High‐GC content regions in the genome have been shown to interfere with great potential to impact human complex traits and diseases.^[^ ^30^ ^]^ Therefore, enhancing PAM recognition space of Cas12a, particularly for GC‐rich PAMs, is crucial for its application in manipulating and investigating the human genome. The engineered ErCas12a variant (enErCas12a) described herein was developed to recognize GC‐rich PAMs as well as other non‐ canonical PAMs, further broadening the targeting scope of Cas12a nucleases, especially for GC‐rich PAM targets. This flexibility will simplify the selection of target sites, generating predictable indels or placing the base‐editing window at the desired nucleotide position in genes associated with immunological diseases, such as ulcerative colitis, inflammatory bowel disease, and celiac disease contains, where Cas nucleases with GC‐rich PAMs are necessary for efficient targeting.^[^ ^30^ ^]^ Considering that activity is a crucial factor in the application of Cas nucleases, we introduced the F840L mutation to improve the editing efficacy of enErCas12a. However, this enhancement came with the trade‐off of increased off‐target effects, potentially resulting from an increased rate of RNP complex formation or improved overall stability of the RNP complex. These findings highlight the balance between editing efficiency and specificity, necessitating careful monitoring to mitigate potential unintended consequences. Moving forward, our research will prioritize strategies that enhance efficiency without compromising specificity, thereby guiding the development of more precise genome editing tools.

Apart from PAM compatibility, the selection of Cas enzymes also involves considering the trade‐off between broader target range and increased off‐target effects. Interestingly, enErCas12a maintains high specificity while expanding PAM recognition, even showing reduced off‐target effects at sites with non‐canonical PAMs, which may be attributed to the stringent matching process required by Cas12a during target cleavage. Recent studies have proposed a strategy for nucleic acid detection using non‐canonical PAM sequences to lower the rates of both on‐target and off‐target cleavage by Cas12a, presenting a promising avenue for enhancing CRISPR‐based nucleic acid detection methods.^[^ ^47^ ^]^ Thus, the efficacy and precision of enErCas12a in targeting sites with non‐canonical PAMs may facilitate the development of additional nucleic acid detection techniques, expanding the detection locations while minimizing false positives, even in the presence of a single‐point mutation. In conclusion, enErCas12a could effectively target previously inaccessible PAMs and maintain high specificity, providing a powerful canonical for improving the practicability of Cas12‐based CRISPR technology.

## 4 Experimental Section

### Plasmids and Oligonucleotides

DNA sequences used in this study can be found in the Table S4 (Supporting Information). The bacterial ErCas12a/crRNA expression plasmid was constructed with the T7 promoter and the J23119 promoter to separately express ErCas12a and the crRNA. A previously described repeat sequence was used for ErCas12a. A synthetic cassette, 5′‐ATGAGAAGAAGAAGGAGGAGGCTACTACTACTA ‐3′, was cloned into the NcoI and BlpI sites of pET28a vector to construct the CreT positive plasmid. The p11‐LacY‐wtx1 plasmid (Addgene #69056) was purchased from Addgene. To generate mutagenized ErCas12a libraries, first NNK saturation mutations were performed for 169, 529, and 535. The resulting mutation library was then used as templates for further mutagenesis. Subsequently, residues 513–677 of ErCas12a were randomly mutagenized, which contain the WED‐II and the PI domain, using Taq DNA Polymerase (M0273S, NEB) at a rate of ≈2 substitutions per kilobase. For the plasmid‐depletion assay, two randomized PAM libraries (each with a different protospacer sequence) were constructed through PCR to add NNNN/NNNNN nucleotides adjacent to the 5′ end of the protospacer. For bacterial genome editing, the pCas12a plasmids were constructed by replacing the cas9 sequence on the pCas plasmid (Addgene #62225) with the cas12a sequence. The crRNA on the pTargetF plasmid (Addgene #62226) was replaced by the crRNA corresponding to Cas12a to construct the crRNA plasmid pTarget. For mammalian genome editing, human codon‐optimized *cas12a* genes were synthesized and cloned into a mammalian expression vector containing dual NLS sequences and a C‐terminal GFP, under the control of the CMV promoter. Plasmids for U6 expression of crRNAs were assembled using the Golden Gate assembly method. For base editing, ABE8e was inserted into was fused to the N‐terminus of dCas12a to construct ABE expression plasmids. For GUIDE‐seq, *ercas12a/enercas12a/enascas12a‐hf* gene and corresponding crRNA were integrated into a plasmid to construct the pHS‐crRNA‐ErCas12a/enErCas12a/enAsCas12a‐HF plasmids.

### Bacterial‐Based Positive Selection Assay for Evolving ErCas12a Variants

To identify ErCas12a variants capable of targeting GC‐rich PAMs, mutagenized ErCas12a/crRNA plasmid libraries were electroporated into *E. coli* BW25113(DE3) cells containing a mix of CreT plasmids bearing CCCC/GCCC/CGCC/CCGC PAMs. Following a 60 min recovery in SOB media, transformations were plated on LB plates containing ampicillin and 10 mm IPTG. The mutated regions of the surviving colonies were sequenced and subcloned into fresh backbone plasmid to perform the negative selection, which were screened on LB plates containing ampicillin and kanamycin. The cleavage efficiency of the mutants was estimated by calculating the survival frequency of colonies on plates containing ampicillin and IPTG divided by the sum of colonies on plates containing ampicillin and IPTG and colonies on plates containing ampicillin and kanamycin. Mutations from survival colonies were visualized using Weblogo.

### PAM Plasmid Depletion Assay

Each randomized NNNN/NNNNN PAM library was electroporated into *E. coli* BW25113 (DE3) cells containing the ErCas12a/crRNA expression system with a spacer targeting the PAM library. Inactive ErCas12a was used as a control. Following a 60 min recovery in SOB media, transformations were plated on LB plates containing ampicillin and kanamycin. Surviving colonies were harvested and plasmids were extracted using the HighPure Maxi Plasmid Kit (Tiangen, China). The resulting plasmid libraries were amplified by PCR with primers containing barcodes, and the PCR products were purified using the E.Z.N.A Gel Extraction Kit (Omega) for deep sequencing. Sequencing reads were sorted using barcodes and analyzed for the corresponding spacer region. The abundances of 256/1024 PAMs were tallied from the aligned reads and normalized by the total reads. The post‐selection PAM depletion value (PPDV) was determined by calculating the ratio of the abundance in the post‐selection group compared to the control group. The mean PPDV<0.1 indicates that the PAMs depletion ratios were over ten‐fold. The PPDVs of wild‐type and variants for PAM depletion experiments with two different spacers are listed in Table S1 (Supporting Information).

### Bacterial Genome Targeting and Editing

The bacterial genome editing assay was conducted in accordance with previously established methods. Briefly, 100 ng of pTarget plasmid containing a genome‐targeting spacer and 300 ng of donor DNA were electroporated into the *E. coli* cells carrying the pCas12a plasmid. Following resuscitation for 1 h, the cells were plated on LB agar supplemented with kanamycin and spectinomycin. After overnight incubation at 30 °C, gene‐editing events in the resulting colonies were verified by PCR amplification and sanger sequencing of the region around the target locus. The targets and PAMs used are detailed in Table S3 (Supporting Information).

### Cell Culture

HEK293T cells (ATCC) were cultured in DMEM supplemented with 10% FBS and 1 × penicillin streptomycin. Cells were cultured at 37 °C with 5% CO_2_ and were regularly passaged when reaching 80% confluency.

### Transfection and DNA Extraction

HEK293T cells (1 × 10^5^) were seeded into 24‐well plates. 12–15 h after plating, cells were transfected with 0.85 µL of jetOPTIMUS reagent (Polyplus‐transfection) using 350 ng of ErCas12a plasmid, 250 ng of crRNA plasmid. Twenty‐four hours after transfection, 4 µg mL^−1^ puromycin was added to the medium. The transfected cells were incubated for another 3 days. Prior to undergoing deep sequencing analyses, the cells were washed with PBS, and the extraction of genomic DNA entailed the introduction of 40 µL of lysis buffer (ThermoFisher Scientific) supplemented with 25 µg mL^−1^ proteinase K. Subsequently, the mixture was incubated at 55 °C for 5 min and then inactivated at 98 °C for 5 min. The targets and PAMs used in HEK293T cells are detailed in Table S2 (Supporting Information).

### High‐Throughput Sequencing

Platinum Direct PCR Universal Master Mix (ThermoFisher Scientific) was used for amplification of target loci sequences in HEK293T cells. The targeted regions (180–250 bp) of interest were amplified via PCR using indicated primers. Forward and reverse barcodes were incorporated at the ends of the PCR products to enable high‐throughput DNA sequencing. Equal amounts of PCR product were pooled and purified with the E.Z.N.A Gel Extraction Kit (Omega). The purified products were sequenced commercially using the NovaSeq platform (GENEWIZ, China). The combined deep sequencing data were separated according to their barcodes and analyzed as previous report.^[^ ^48^ ^]^ The editing efficiency was determined by dividing the indel reads by the total reads.

### GUIDE‐Seq

GUIDE‐seq experiments were performed as previously described.^[^ ^49^ ^]^ Briefly, 5 × 10^5^ HEK293T cells were transfected with 2.5 µg of ErCas12a/enErCas12a expression plasmid and 350 ng of double‐stranded oligodeoxynucleotide (dsODN). Cells were collected 72 h after puromycin selection for genomic extraction. The genome library was prepared and subjected for high‐throughput sequencing commercially.

## Conflict of Interest

The authors declare no conflict of interest.

## Author Contributions

Z.C. and J.X. contributed equally to this work. B.W. and M.L. supervised the project. Z.H.C., J.Y.X., and Z.Y.W. performed biological experiments, J.Y.S. analyzed the high‐throughput sequencing results, Y.L.C. and T.Z. analyzed the data, H.Y.Y. guided the cell experiment. Z.H.C. and B.W. drafted the manuscript, which was revised and approved by all authors.

## Supporting information



Supporting Information

## Data Availability

The data that support the findings of this study are available in the supplementary material of this article.
