# Supplementary material for: Small RNA Toxin‐Assisted Evolution of GC‐Preferred ErCas12a for Enhanced Genome Targeting Range
Source: Adv Sci (Weinh). 2025 May 20;12(29):e17105. doi: 10.1002/advs.202417105 (PMC12362730; doi:10.1002/advs.202417105)
Supplement: Supplementary file 1 — Supporting Information [file ADVS-12-e17105-s001.docx]

Supporting Information

**Small RNA Toxin-assisted Evolution of GC-preferred ErCas12a for** **Enhanced Genome Targeting Range**

*Zehua Chen^#^, Junyuan Xue^#^, Ziying Wang, Jinyuan Sun, Yinglu Cui, Tong Zhu, Huaiyi Yang, Ming Li,^*^ and Bian Wu^*^*

This PDF includes:

**Figure S1 to S12**

Figure S1. Functional validation of key components in CcdB-based positive system.

Figure S2. Construction of CreT-based positive screening system.

Figure S3. Sequencing results during the process of mutagenesis and screening.

Figure S4. PAM specificities of ErCas12a-RRNNRR and ErCas12a-RRNNRRQ variants.

Figure S5. Comparison of the targeting efficiencies between AsCas12a, enAsCas12a, LbCas12a, impLbCas12a, ErCas12a, and enErCas12a across 66 PAMs, evaluated through the genome interference assay.

Figure S6. Editing efficiency of wild type and the enErCas12a variant in *E. coli* genome.

Figure S7. Comparison of editing efficiency by AsCas12a, enAsCas12a, LbCas12a, impLbCas12a, ErCas12a and enErCas12a in the HEK293T genome.

Figure S8. Comparison of editing efficiency by enAsCas12a, impLbCas12a and enErCas12a in the Hela genome.

Figure S9. Structural differences between WT ErCas12a and enErCas12a.

Figure S10. Genome editing with enErCas12a and enErCas12a-F840L at endogenous loci in HEK293T cells.

Figure S11. Comparison of editing efficiency by enErCas12a and enErCas12a-F840L in the HEK293T genome.

Figure S12. Analysis of ErCas12a variants specificity in HEK293T cells.

Figure S13. Characterization of enErCas12a specificity.

**Table S3 to S4**

Table S3. Target sites with various PAMs for gene editing in *E. coli*.

Table S4. Sequences of promoters, RBS and representative genes used in this study.


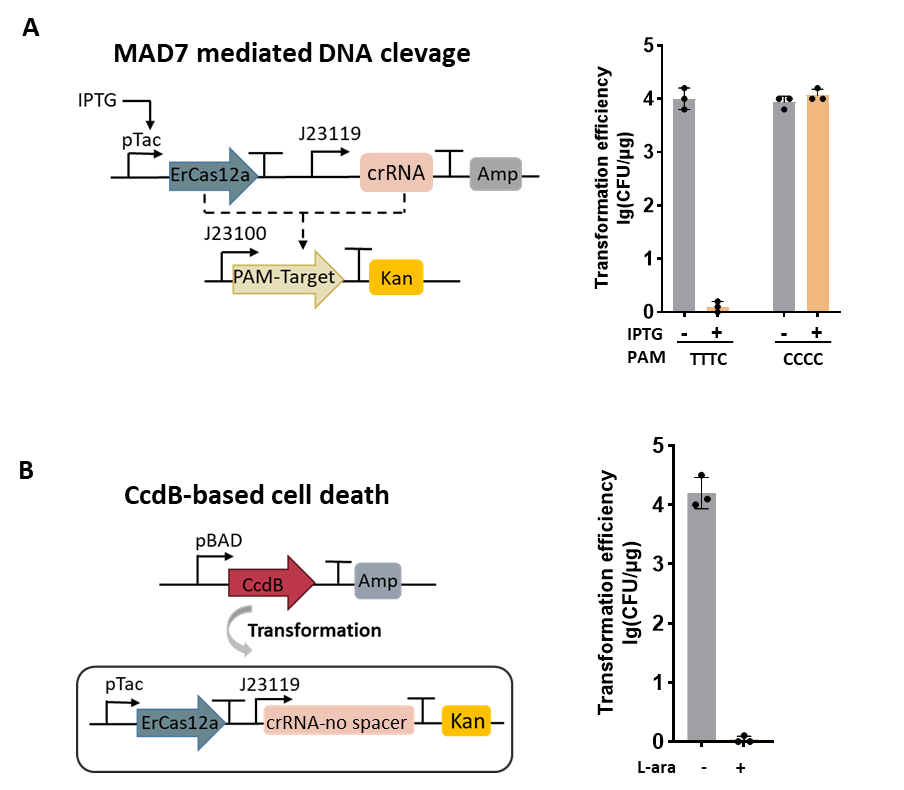


**Figure S1.** Functional validation of key components in CcdB-based positive system. **A.** Plasmid elimination mediated by ErCas12a in *E. coli*. The ErCas12a /crRNA expression plasmid, designed to target a site bearing PAM TTTC or PAM CCCC, along with the reporter plasmid containing Kan-resistance, were introduced into *E.coli* BW25113 (DE3). Subsequently, equimolar quantities of cells were seeded onto plates supplemented with ampicillin + kanamycin (IPTG -) and plates with ampicillin + kanamycin + 10 mM IPTG (IPTG +). These cells could form healthy colonies on plates (IPTG -) but lack the ability to form colonies on plates (IPTG +), suggesting that ErCas12a/crRNA could effectively target and cut DNA sequences containing PAM TTTC, but not PAM CCCC. Data are mean ± s. d. of n = 3 independent experiments. **B.** Verification assay for CcdB cytotoxicity. The p11-LacY-wtx1 plasmid was introduced into *E. coli* BW25113 (DE3) containing the ErCas12a/crRNA plasmid, then equimolar quantities of cells were seeded onto plates supplemented with ampicillin + kanamycin (L-ara -) and plates with ampicillin + kanamycin +10 mM L-ara (L-ara +). These cells could form healthy colonies on plates (L-ara -) but lack the ability to form colonies on plates (L-ara +), suggesting that the expression of CcdB induced by L-ara could lead to cell death. Data are mean ± s. d. of n = 3 independent experiments.


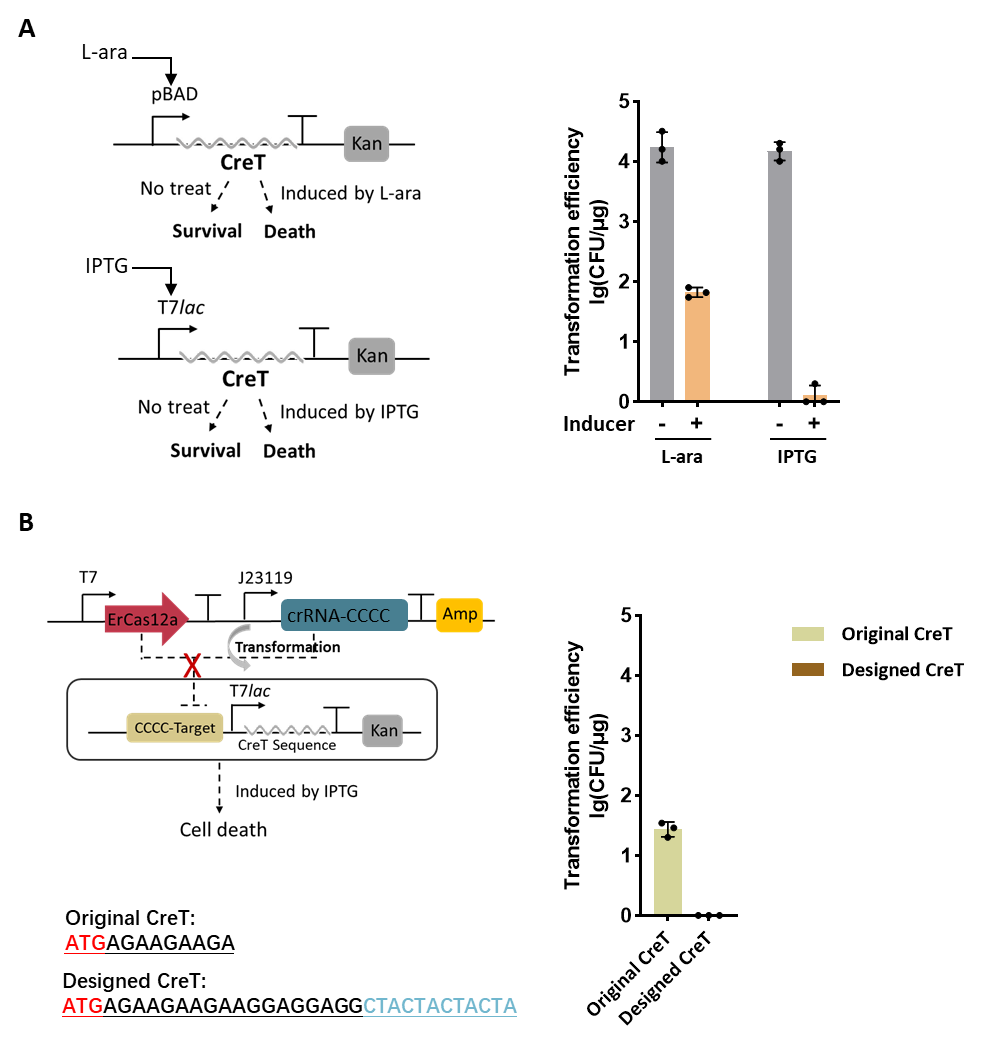


**Figure S2.** Construction of CreT-based positive screening system. **A.** Construction of CreT inducible expression system. The CreT plasmid controlled by either the T7*lac* promoter or the araBAD promoter was introduced into *E. coli* BW25113 (DE3). Equal quantities of bacteria were spread onto plates with (+) and without (-) inducers. These cells could form colonies on kanamycin plates but failed to form colonies on plates containing 10 mM IPTG, suggesting that the CreT RNA effectively cause cell death upon induced by IPTG. Data are mean ± s. d. of n = 3 independent experiments. **B.** Optimization of CreT sequence. The ErCas12a/crRNA expression plasmid, specifically engineered to target a site bearing PAM CCCC, was introduced into *E. coli* BW25113(DE3) containing the CreT plasmid encoding either an original CreT or a modified version. Subsequently, equal amounts of cells were plated on agar plates supplemented with ampicillin and 10 mM IPTG. The outcomes of the experiment revealed that the designed CreT sequence effectively enhanced the activity of CreT toxin upon induction. CreT sequences were provided with underline. initiation codon ATG was marked in blue, AGA/AGG was marked in black, CTA was marked in blue. Data are mean ± s. d. of n = 3 independent experiments.


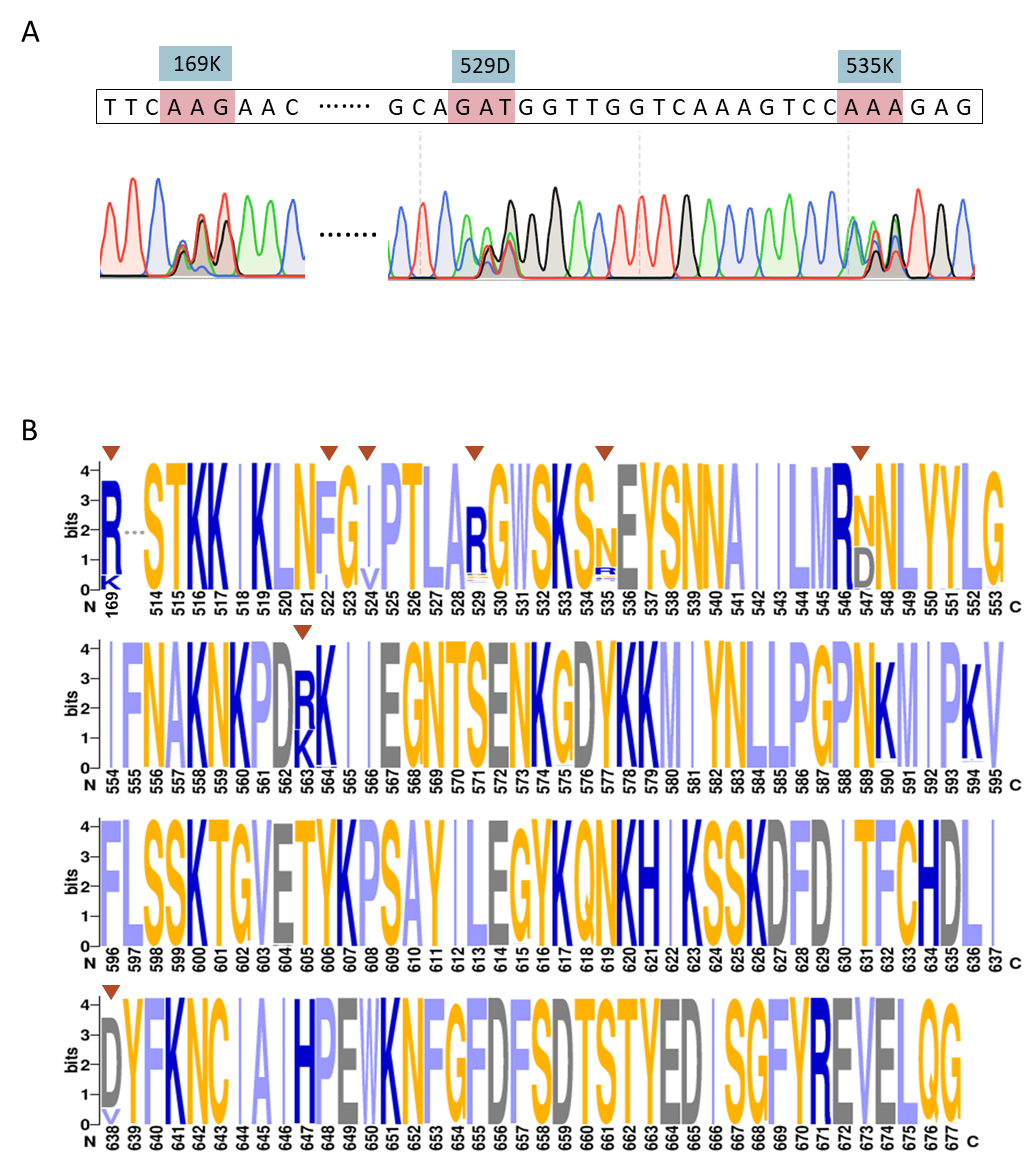


**Figure S3.** Sequencing results during the process of mutagenesis and screening. **A.** Sequencing peak of the mixed plasmid library which was used as templates before random mutagenesis. **B.** Statistical diagram of the amino acid sequences of the mutant region of survived bacteria from the positive screening in the first-round evolution. About 400 clones were selected for sequencing. The most frequent substitutions were marked with red triangles.


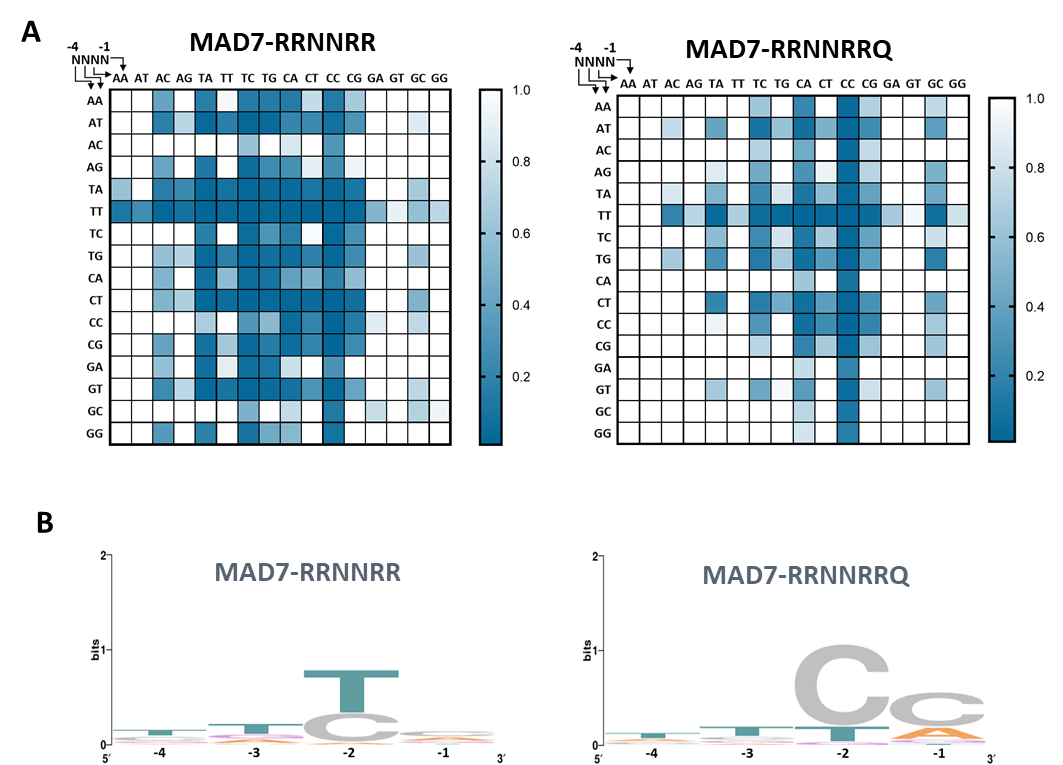


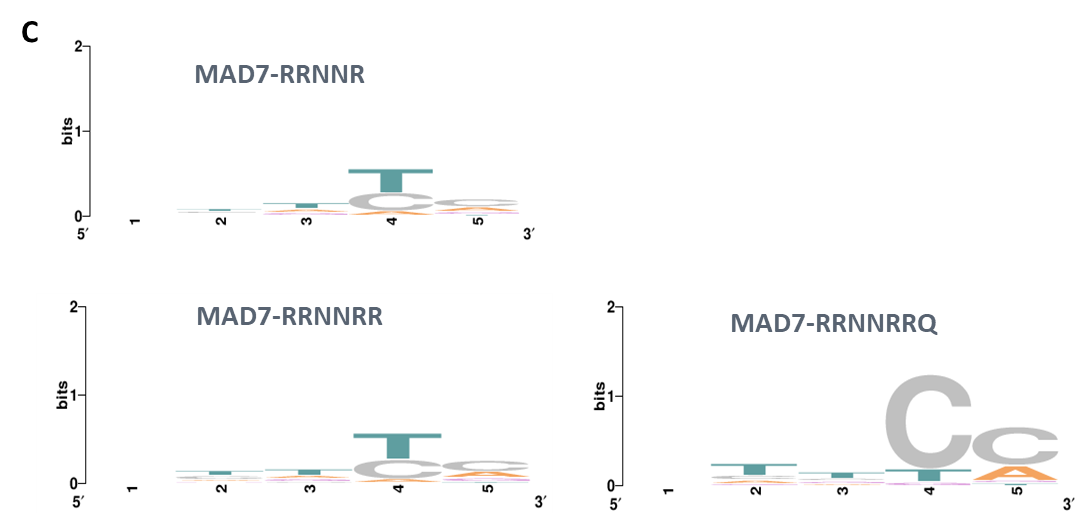


**Figure S4.** PAM specificities of ErCas12a-RRNNRR and ErCas12a-RRNNRRQ variants. **A.** PAM preference profiles, assessed by mean PPDVs, for ErCas12a-RRNNRR and ErCas12a-RRNNRRQ variants. **B.** Web logos of the most depleted NNNN PAMs (with a mean of PPDVs smaller than 0.1) for ErCas12a-RRNNRR and ErCas12a-RRNNRRQ variants. **C.** Web logos of the most depleted NNNNN PAMs (with a mean of PPDVs smaller than 0.1) for ErCas12a -RRNNR, ErCas12a-RRNNRR and ErCas12a-RRNNRRQ variants.


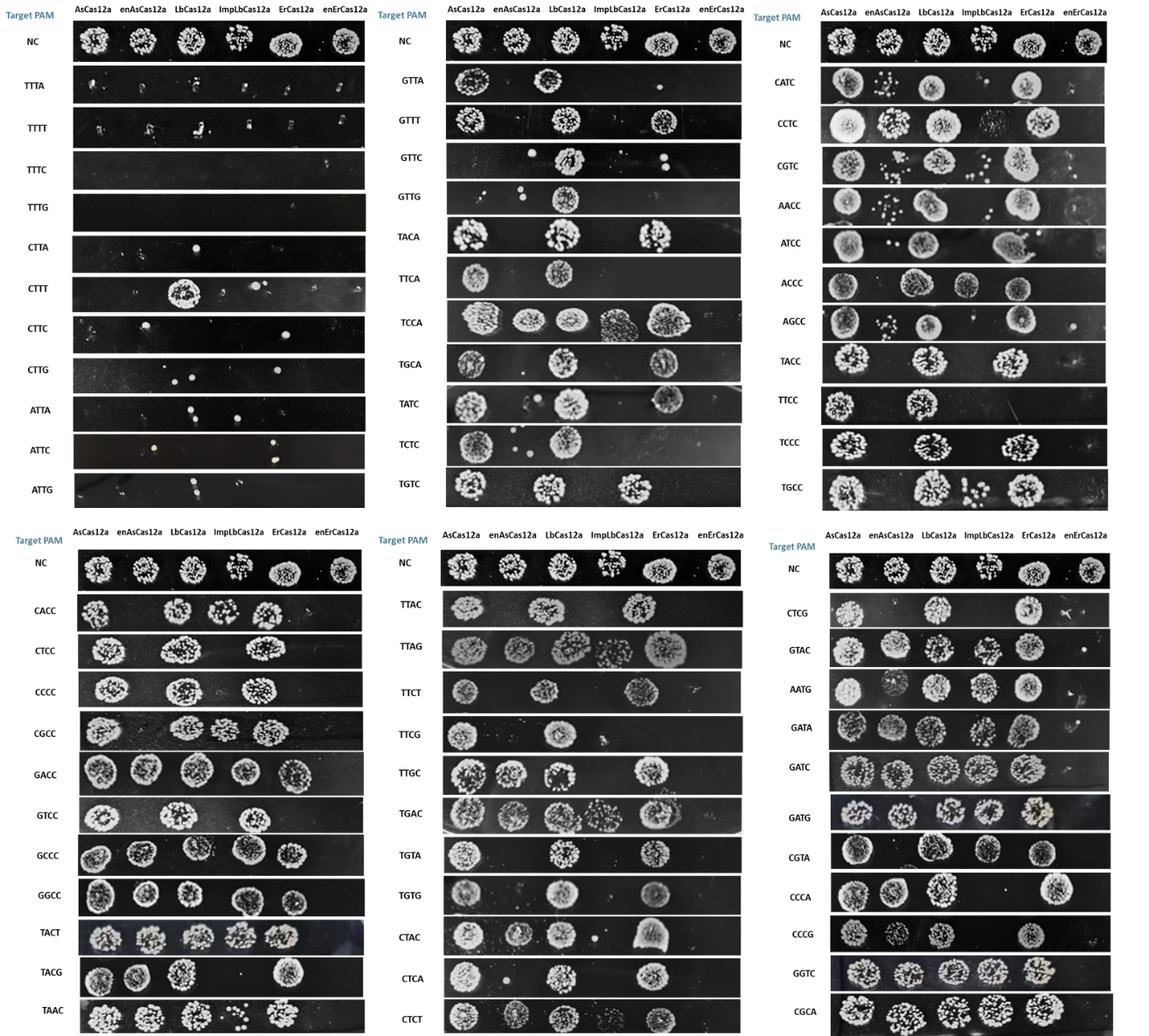


**Figure S5.** Comparison of the targeting efficiencies between AsCas12a, enAsCas12a, LbCas12a, impLbCas12a, ErCas12a, and enErCas12a across 66 PAMs, evaluated through the genome interference assay. The PAM sequences were shown on the left. "NC" represent pTarget plasmid without target site.


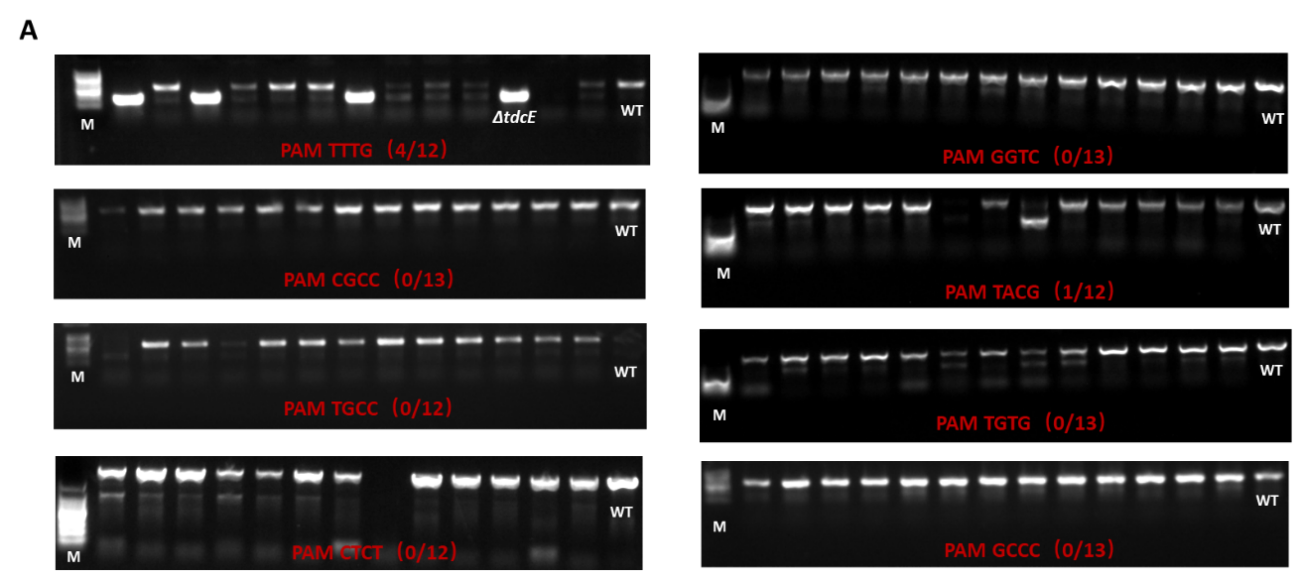


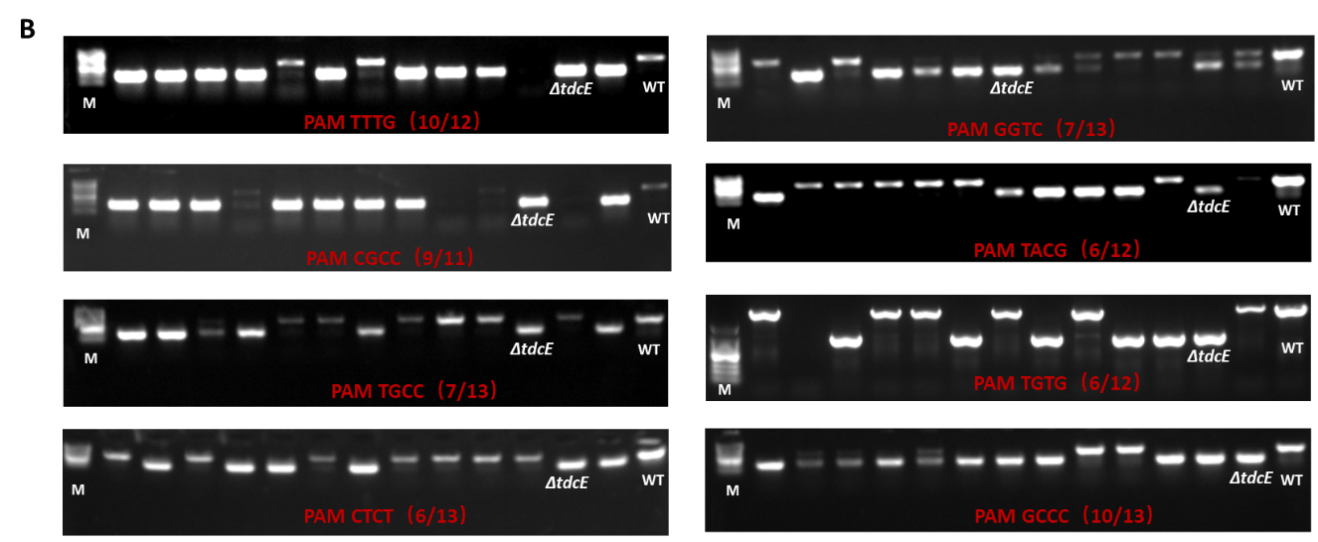


**Figure S6.** Editing efficiency of wild type and the enErCas12a variant in *E. coli* genome. **A-B.** Deletion of *tdcE* in *E. coli* using wild type (**A**) and the enErCas12a variant (**B**) at 8 target sites bearing different PAMs. PAM sequences were marked in red.


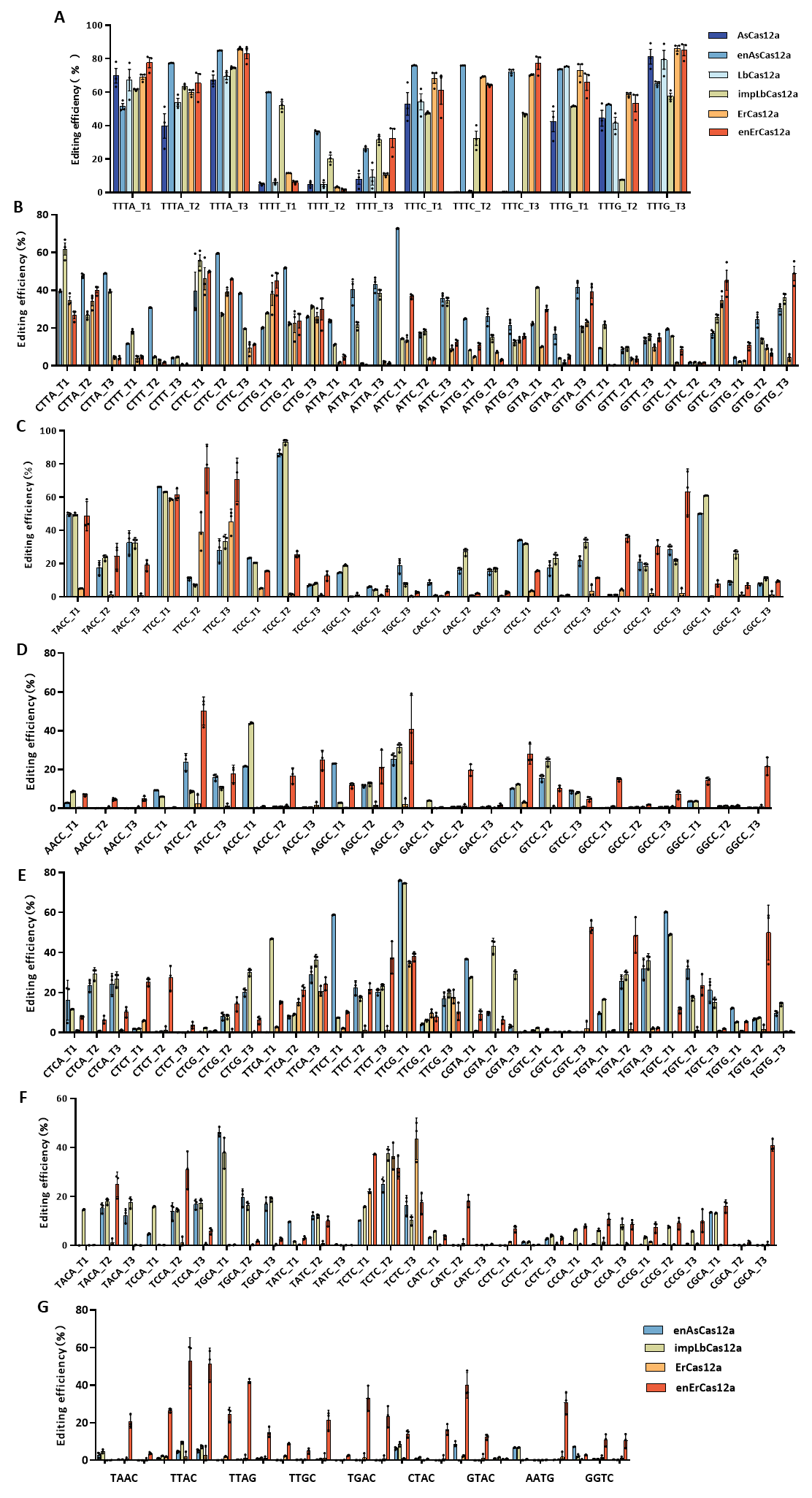


**Figure S7.** Comparison of editing efficiency by AsCas12a, enAsCas12a, LbCas12a, impLbCas12a, ErCas12a and enErCas12a in the HEK293T genome. **A**. Indel frequencies generated by AsCas12a, enAsCas12a, LbCas12a, impLbCas12a, ErCas12a and enErCas12a at target sites with TTTN. **B-G.** Indel frequencies generated by enAsCas12a, impLbCas12a, ErCas12a and enErCas12a at target sites with VTTN (**B**), YNCC (**C**), YTCD/YGTV (**D**), TVCA/YMTC **(E)** and other non-candidate (**E-G**) PAMs. Three days after plasmid transfection and puromycin selection, genomic DNA was extracted for targeted deep sequencing. The PAM for each site is indicated below. Data are mean ± s.d. of n = 3 independent experiments.


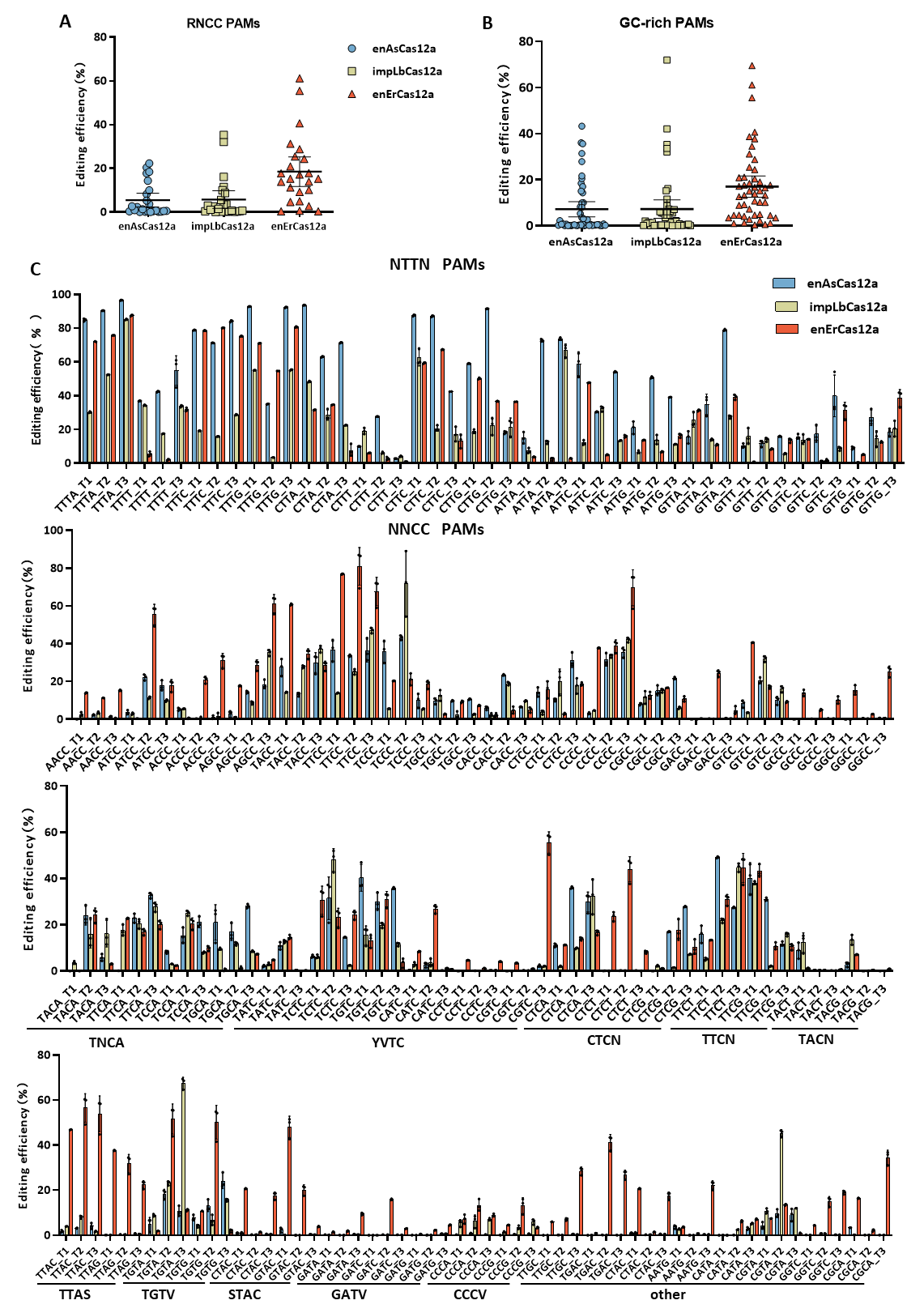


**Figure S8.** Comparison of editing efficiency by enAsCas12a, impLbCas12a and enErCas12a in the Hela genome. **A-B.** Summary of the editing efficiencies mediated by the enAsCas12a, impLbCas12a and enErCas12a at targets with RNCC PAMs **(A)** and GC-rich PAMs (the GC content in the PAM sequences exceeded 75%) **(B)**. Data are mean ± s. d. of n = 3 independent experiments. **C.** Comparison of editing efficiency by enAsCas12a, impLbCas12a and enErCas12a.


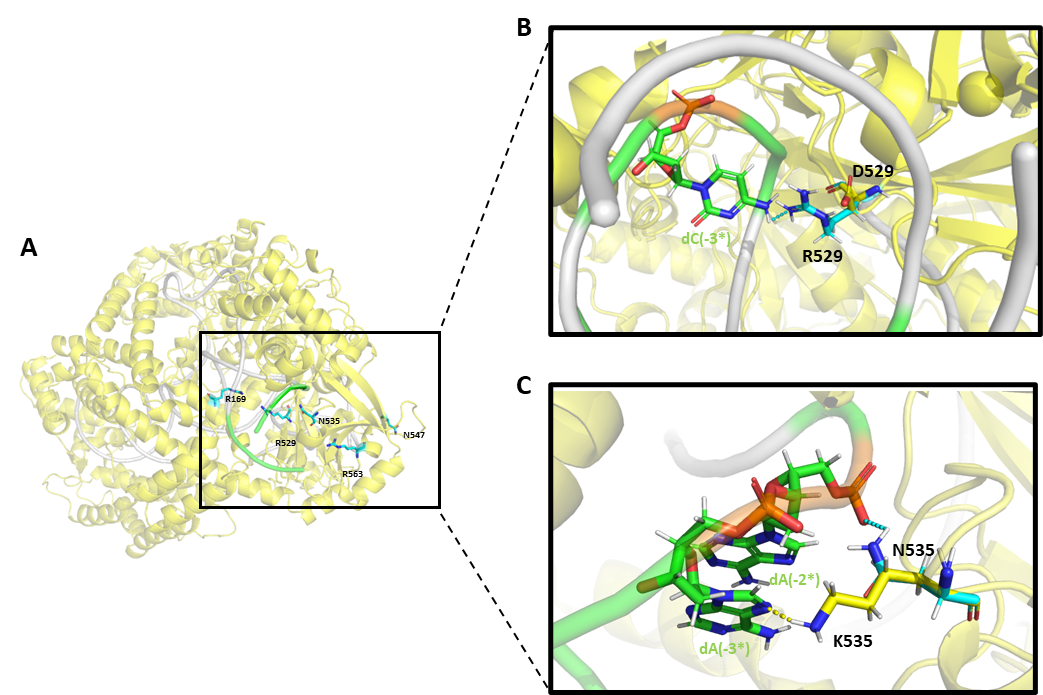


**Figure S9.** Structural differences between WT ErCas12a and enErCas12a. **A.** Overall structural model of enErCas12a predicted by AlphaFold3. Mutated amino acids are shown in blue. PAM nucleotides are marked as green. **B.** Structural differences in D529 and R529 interacting with PAM-complementary nucleotide between WT and enErCas12a. The substituted residues are highlighted by blue labels. **C.** Structural differences in K535 and N535interacting with PAM-complementary nucleotide between WT and enErCas12a. The substituted residues are highlighted by blue labels. The structural figures were prepared using PYMOL (http://pymol.org).


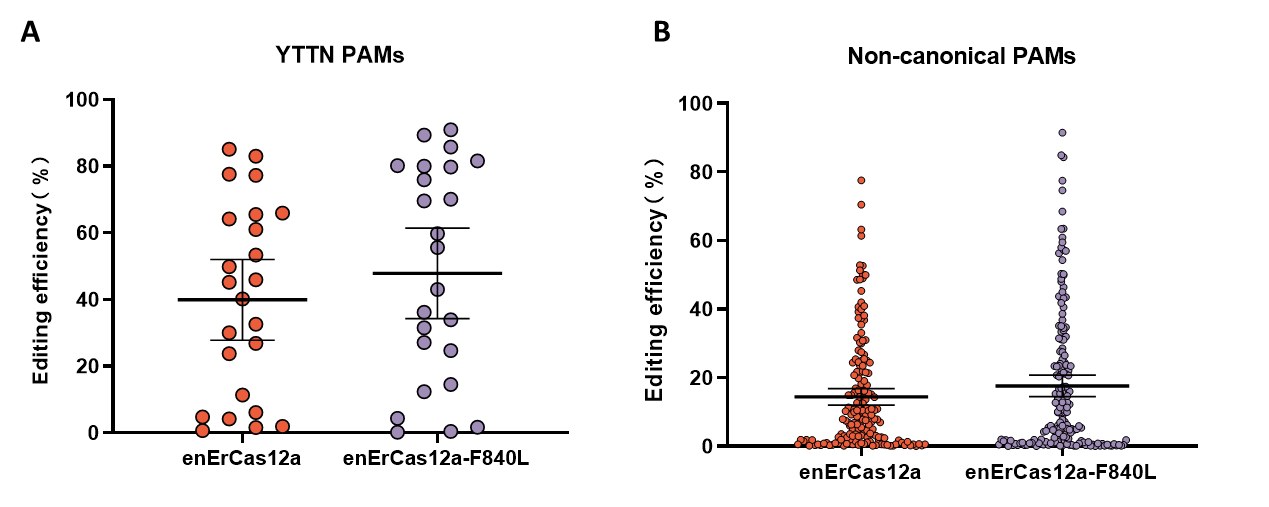


**Figure S10.** Genome editing with enErCas12a and enErCas12a-F840L at endogenous loci in HEK293T cells. **A-B.** Summary of the indel frequencies mediated by enErCas12a and enErCas12a-F840L at 198 endogenous target sites with original YTTN PAMs **(A)** or non-canonical PAMs **(B)** in HEK293T cells. Data are mean ± s. d. of n = 3 independent experiments.


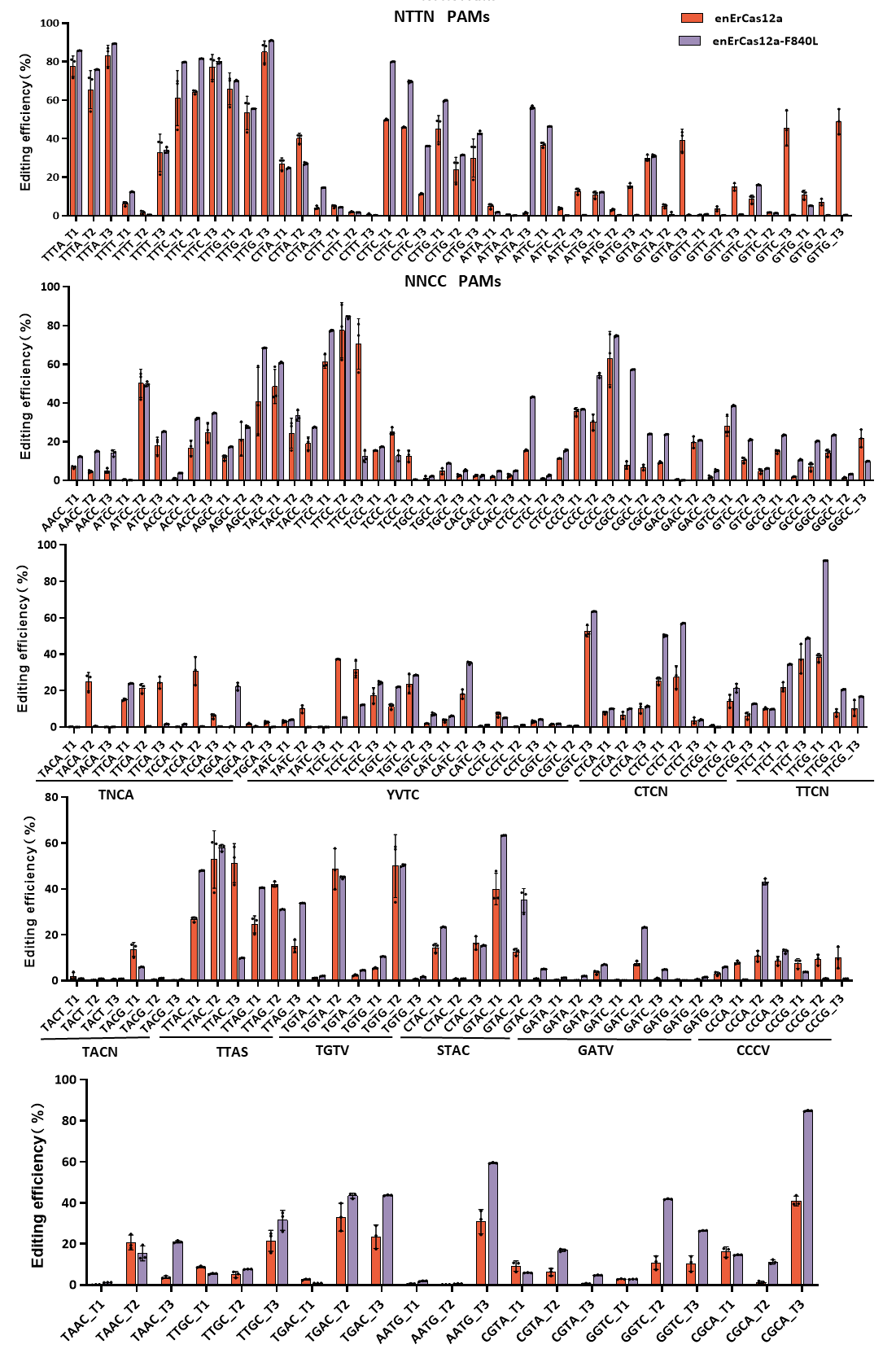


**Figure S11.** Comparison of editing efficiency by enErCas12a and enErCas12a-F840L in the HEK293T genome. Data are mean ± s. d. of n = 3 independent experiments.

**
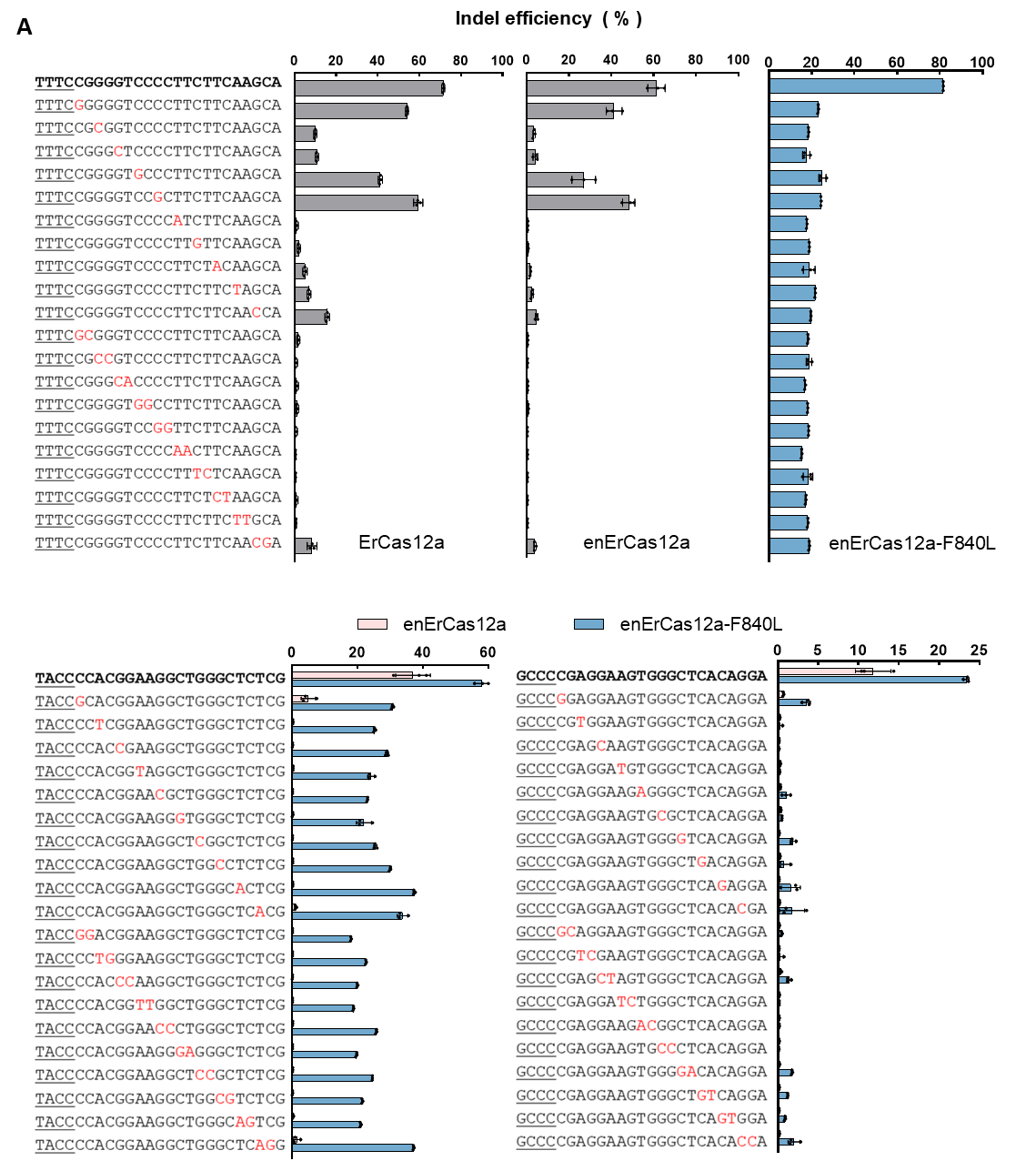
**

**Figure S12.** Analysis of ErCas12a variants specificity in HEK293T cells. **A.** Mismatch tolerance of WT ErCas12a, enErCas12a and enErCas12a-F840L at the target site containing PAM TTTC in HEK293T cells. **B.** Mismatch tolerance of enErCas12a and enErCas12a-F840L for the target sites containing PAM TACC (left) and PAM GCCC (right) in HEK293T cells. Mismatched crRNAs that differed from the target sites by 1 or 2 nt were tested in HEK293T cells. Mismatched bases were shown in red and PAM sequences were marked with underscores. The mean and s. d. of all individual values of n = 3 independent biological replicates were plotted.


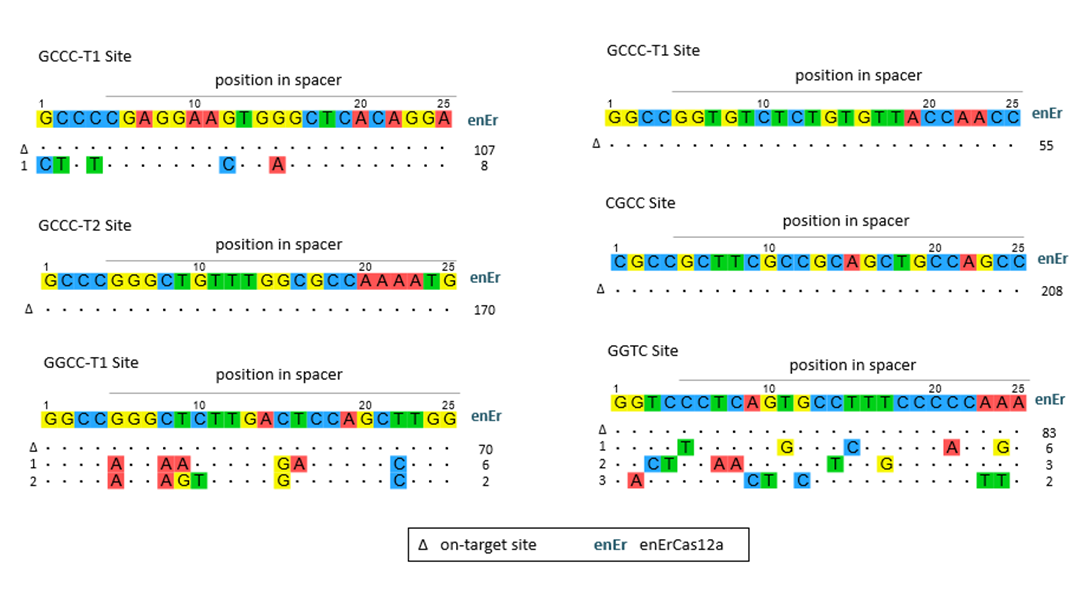


**Figure S13**. Characterization of enErCas12a specificity. GUIDE-seq genome-wide specificity profiles for enErCas12a on sites with GC-rich PAMs. Mismatched positions in off-target sites were highlighted in colour and GUIDE-seq read counts were shown to the right of the sequences.

**Table S3.** **Target sites with various PAMs for gene targeting and editing in *E. coli***

| Sequences Description | 5’ 🡪 3’ |
| --- | --- |
| Target site for PAM TTTG | GTCGGAAGAATTACCGATTGC |
| Target site for PAM TTTA | AAGCTGGCGTACTCAATGAGC |
| Target site for PAM TTTC | GGCCCGACCTGAATCTTCAGC |
| Target site for PAM TTTT | GCGATTTCACTGCCGCCAGAT |
| Target site for PAM CTTA | TCTGGCGGCAGTGAAATCGCA |
| Target site for PAM CTTT | GGTGCACGCGCTAACCTGGCG |
| Target site for PAM CTTC | CTGCGTACACCGGAATTTGAT |
| Target site for PAM CTTG | TCGTGCATGTAGTGAATGATA |
| Target site for PAM ATTA | CCGATTGCCTTCAAAAAATAT |
| Target site for PAM ATTC | AGGTCGGGCCGAAAACAGCAC |
| Target site for PAM ATTG | CGTAGAGCAGCGTTTTCGCCA |
| Target site for PAM GTTA | ATTGCGTAGAGCAGCGTTTTC |
| Target site for PAM GTTT | TCGGCCCGACCTGAATCTTCA |
| Target site for PAM TACA | CCGGAATTTGATTCGCTGTTC |
| Target site for PAM TTCA | AAAAATATGCCGCGCAGGTGT |
| Target site for PAM TCCA | CCCCGCCGTTAATTGCGTAGA |
| Target site for PAM TGCA | CGCGCTAACCTGGCGAAAACG |
| Target site for PAM TATC | TACATTGAGCGCGACTTTAAA |
| Target site for PAM TCTC | CGGCGACCCAATCTGGGCGAC |
| Target site for PAM TGTC | GATCGTCACCTCTTCCTTGCA |
| Target site for PAM CATC | AGATCGTCATTTTCATACTGC |
| Target site for PAM AACC | TGACCATTCTTTGGTCGGAAG |
| Target site for PAM ATCC | GTATGGTACGCTTCCTGCGTA |
| Target site for PAM TACC | GATTGCCTTCAAAAAATATGC |
| Target site for PAM TTCC | GCTATTTGCACACCCTGCACA |
| Target site for PAM TAAC | CTGACCATTCTTTGGTCGGAA |
| Target site for PAM GTTC | TTTGGTGCACGCGCTAACCTG |
| Target site for PAM TTAG | GTTCCGGTGCCGGCCCCATAG |
| Target site for PAM TTGC | AGTATGAAAATGACGATCTGA |
| Target site for PAM GTAC | TGACTTCAACAGCGACGATTA |
| Target site for PAM AATG | ATATTCAGCGCGCTGATGTAC |
| Target site for PAM GATA | TCTACATTGAGCGCGACTTTA |
| Target site for PAM GATC | CGTATGGTACGCTTCCTGCGT |
| Target site for PAM GATG | CGTACTGACTTCAACAGCGAC |
| Target site for PAM CGTA | CTGACTTCAACAGCGACGATT |
| Target site for PAM TGAC | CAAAAACTCCTTCCGCTATTT |
| Target site for PAM GTTG | AAGTCAGTACGCATCAGATCG |
| Target site for PAM CGCC | CGGCGCAGAATGCGCAGGAAG |
| Target site for PAM TGCC | TTCAAAAAATATGCCGCGCAG |
| Target site for PAM CTCT | TCCTTGCAGTATGAAAATGAC |
| Target site for PAM GGTC | GGAAGAATTACCGATTGCCTT |
| Target site for PAM TACG | CGATTGCCTGCTGCGTCAGCC |
| Target site for PAM TGTG | CCTGCTGCTCATTGAGTACGC |
| Target site for PAM GCCC | AGATTGGGTCGCCGGAGAACA |
| Target site for PAM CCTC | GATATCTACATTGAGCGCGAC |
| Target site for PAM CGTC | CAGCCCCATCCCGCCGATCAC |
| Target site for PAM ACCC | AATCTGGGCGACGGAAGTGAT |
| Target site for PAM AGCC | ATATTTCGCCGCCATTTCCTG |
| Target site for PAM TCCC | GCCGATCACTTCCGTCGCCCA |
| Target site for PAM GACC | CAATCTGGGCGACGGAAGTGA |
| Target site for PAM CACC | GGAATTTGATTCGCTGTTCTC |
| Target site for PAM CTCC | GGCGACCCAATCTGGGCGACG |
| Target site for PAM CCCC | ATAGTGTGCAGGGTGTGCAAA |
| Target site for PAM GTCC | AGCCCCATCCCGCCGATCACT |
| Target site for PAM GGCC | CGACCTGAATCTTCAGCTTCT |
| Target site for PAM CTCG | TCCACCCCGCCGTTAATTGCG |
| Target site for PAM CCCA | ATGGTGATTGGTAAGCAAATG |
| Target site for PAM CCCG | ACCTGAATCTTCAGCTTCTCG |
| Target site for PAM CGCA | GGTGTCGATCGTCACCTCTTC |

**Table S4. Sequences of promoters, RBS and representative genes used in this study.**

**pT7-ErCas12a-J23119-crRNA**

pT7 promoter in red, ErCas12a in green, J23119 promoter in blue, repeat of crRNA for positive screening in orange with repeat unlined.

TAATACGACTCACTATAGGTCAGCAGGACGCAAAGAGGAGAAACTGACCATGAACAACGGCACAAATAATTTTCAGAACTTCATCGGGATCTCAAGTTTGCAGAAAACGCTGCGCAATGCTCTGATCCCCACGGAAACCACGCAACAGTTCATCGTCAAGAACGGAATAATTAAAGAAGATGAGTTACGTGGCGAGAACCGCCAGATTCTGAAAGATATCATGGATGACTACTACCGCGGATTCATCTCTGAGACTCTGAGTTCTATTGATGACATAGATTGGACTAGCCTGTTCGAAAAAATGGAAATTCAGCTGAAAAATGGTGATAATAAAGATACCTTAATTAAGGAACAGACAGAGTATCGGAAAGCAATCCATAAAAAATTTGCGAACGACGATCGGTTTAAGAACATGTTTAGCGCCAAACTGATTAGTGACATATTACCTGAATTTGTCATCCACAACAATAATTATTCGGCATCAGAGAAAGAGGAAAAAACCCAGGTGATAAAATTGTTTTCGCGCTTTGCGACTAGCTTTAAAGATTACTTCAAGAACCGTGCAAATTGCTTTTCAGCGGACGATATTTCATCAAGCAGCTGCCATCGCATCGTCAACGACAATGCAGAGATATTCTTTTCAAATGCGCTGGTCTACCGCCGGATCGTAAAATCGCTGAGCAATGACGATATCAACAAAATTTCGGGCGATATGAAAGATTCATTAAAAGAAATGAGTCTGGAAGAAATATATTCTTACGAGAAGTATGGGGAATTTATTACCCAGGAAGGCATTAGCTTCTATAATGATATCTGTGGGAAAGTGAATTCTTTTATGAACCTGTATTGTCAGAAAAATAAAGAAAACAAAAATTTATACAAACTTCAGAAACTTCACAAACAGATTCTATGCATTGCGGACACTAGCTATGAGGTCCCGTATAAATTTGAAAGTGACGAGGAAGTGTACCAATCAGTTAACGGCTTCCTTGATAACATTAGCAGCAAACATATAGTCGAAAGATTACGCAAAATCGGCGATAACTATAACGGCTACAACCTGGATAAAATTTATATCGTGTCCAAATTTTACGAGAGCGTTAGCCAAAAAACCTACCGCGACTGGGAAACAATTAATACCGCCCTCGAAATTCATTACAATAATATCTTGCCGGGTAACGGTAAAAGTAAAGCCGACAAAGTAAAAAAAGCGGTTAAGAATGATTTACAGAAATCCATCACCGAAATAAATGAACTAGTGTCAAACTATAAGCTGTGCAGTGACGACAACATCAAAGCGGAGACTTATATACATGAGATTAGCCATATCTTGAATAACTTTGAAGCACAGGAATTGAAATACAATCCGGAAATTCACCTAGTTGAATCCGAGCTCAAAGCGAGTGAGCTTAAAAACGTGCTGGACGTGATCATGAATGCGTTTCATTGGTGTTCGGTTTTTATGACTGAGGAACTTGTTGATAAAGACAACAATTTTTATGCGGAACTGGAGGAGATTTACGATGAAATTTATCCAGTAATTAGTCTGTACAACCTGGTTCGTAACTACGTTACCCAGAAACCGTACAGCACGAAAAAGATTAAATTGAACTTTGGAATACCGACGTTAGCAGATGGTTGGTCAAAGTCCAAAGAGTATTCTAATAACGCTATCATACTGATGCGCAACAATCTGTATTATCTGGGCATCTTTAATGCGAAGAATAAACCGGACAGGAAGATTATCGAGGGTAATACGTCAGAAAATAAGGGTGACTACAAAAAGATGATTTATAATTTGCTCCCGGGTCCCAACAAAATGATCCCGAAAGTTTTCTTGAGCAGCAAGACGGGGGTGGAAACGTATAAACCGAGCGCCTATATCCTAGAGGGGTATAAACAGAATAAACATATCAAGTCTTCAAAAGACTTTGATATCACTTTCTGTCATGATCTGATCGACTACTTCAAAAACTGTATTGCAATTCATCCCGAGTGGAAAAACTTCGGTTTTGATTTTAGCGACACCAGTACTTATGAAGACATTTCCGGGTTTTATCGTGAGGTAGAGTTACAAGGTTACAAGATTGATTGGACATACATTAGCGAAAAAGACATTGATCTGCTGCAGGAAAAAGGTCAACTGTATCTGTTCCAGATATATAACAAAGATTTTTCGAAAAAATCAACCGGGAATGACAACCTTCACACCATGTACCTGAAAAATCTTTTCTCAGAAGAAAATCTTAAGGATATCGTCCTGAAACTTAACGGCGAAGCGGAAATCTTCTTCAGGAAGAGCAGCATAAAGAACCCAATCATTCATAAAAAAGGCTCGATTTTAGTCAACCGTACCTACGAAGCAGAAGAAAAAGACCAGTTTGGCAACATTCAAATTGTGCGTAAAAATATTCCGGAAAACATTTATCAGGAGCTGTACAAATACTTCAACGATAAAAGCGACAAAGAGCTGTCTGATGAAGCAGCCAAACTGAAGAATGTAGTGGGACACCACGAGGCAGCGACGAATATAGTCAAGGACTATCGCTACACGTATGATAAATACTTCCTTCATATGCCTATTACGATCAATTTCAAAGCCAATAAAACGGGTTTTATTAATGATAGGATCTTACAGTATATCGCTAAAGAAAAAGACTTACATGTGATCGGCATTGATCGGGGCGAGCGTAACCTGATCTACGTGTCCGTGATTGATACTTGTGGTAATATAGTTGAACAGAAAAGCTTTAACATTGTAAACGGCTACGACTATCAGATAAAACTGAAACAACAGGAGGGCGCTAGACAGATTGCGCGGAAAGAATGGAAAGAAATTGGTAAAATTAAAGAGATCAAAGAGGGCTACCTGAGCTTAGTAATCCACGAGATCTCTAAAATGGTAATCAAATACAATGCAATTATAGCGATGGAGGATTTGTCTTATGGTTTTAAAAAAGGGCGCTTTAAGGTCGAACGGCAAGTTTACCAGAAATTTGAAACCATGCTCATCAATAAACTCAACTATCTGGTATTTAAAGATATTTCGATTACCGAGAATGGCGGTCTCCTGAAAGGTTATCAGCTGACATACATTCCTGATAAACTTAAAAACGTGGGTCATCAGTGCGGCTGCATTTTTTATGTGCCTGCTGCATACACGAGCAAAATTGATCCGACCACCGGCTTTGTGAATATCTTTAAATTTAAAGACCTGACAGTGGACGCAAAACGTGAATTCATTAAAAAATTTGACTCAATTCGTTATGACAGTGAAAAAAATCTGTTCTGCTTTACATTTGACTACAATAACTTTATTACGCAAAACACGGTCATGAGCAAATCATCGTGGAGTGTGTATACATACGGCGTGCGCATCAAACGTCGCTTTGTGAACGGCCGCTTCTCAAACGAAAGTGATACCATTGACATAACCAAAGATATGGAGAAAACGTTGGAAATGACGGACATTAACTGGCGCGATGGCCACGATCTTCGTCAAGACATTATAGATTATGAAATTGTTCAGCACATATTCGAAATTTTCCGTTTAACAGTGCAAATGCGTAACTCCTTGTCTGAACTGGAGGACCGTGATTACGATCGTCTCATTTCACCTGTACTGAACGAAAATAACATTTTTTATGACAGCGCGAAAGCGGGGGATGCACTTCCTAAGGATGCCGATGCAAATGGTGCGTATTGTATTGCATTAAAAGGGTTATATGAAATTAAACAAATTACCGAAAATTGGAAAGAAGATGGTAAATTTTCGCGCGATAAACTCAAAATCAGCAATAAAGATTGGTTCGACTTTATCCAGAATAAGCGCTATCTCTAATACTAGTCCAGGCGCTTCTAGAGCTCGGTACCAAATTCCAGAAAAGAGGCCGCGAAAGCGGCCTTTTTTCGTTTTGGTCCTACTAGATGCCTCCACACCGCTCGTCACATCCTGAATTCTAAAGATCTTTGACAGCTAGCTCAGTCCTAGGTATAATACTAGTGTCAAAAGACCTTTTTAATTTCTACTCTTGTAGATCTGCGTTATCCCCTGATTCTG

**pT7lac-CreT**

pT7 promoter in red, lac operator in orange, CreT sequence in green

TAATACGACTCACTATAGGGGAATTGTGAGCGGATAACAATTCCCCTCTAGAAATAATTTTGTTTAACtttaAGAAGGAGATATACCATGAGAAGAAGAAGGAGGAGGCTACTACTACTATGATACTCTGGCTGGCATCTGTCCTTGGAAACACTCATGCCAGCCACGATCAGGGGATCCATAACTAGCATAACCCCTTGGGGCCTCTAAACGGGTCTTGAGGGGTTTTTTGCTGAAAGGAGGAACTATATCCGGAT

**U6-sg-puroR for gene editing in mammalian cells**

U6 promoter in red, repeat of crRNA in orange, hPGK promoter in blue, puroR in green

GAGGGCCTATTTCCCATGATTCCTTCATATTTGCATATACGATACAAGGCTGTTAGAGAGATAATTGGAATTAATTTGACTGTAAACACAAAGATATTAGTACAAAATACGTGACGTAGAAAGTAATAATTTCTTGGGTAGTTTGCAGTTTTAAAATTATGTTTTAAAATGGACTATCATATGCTTACCGTAACTTGAAAGTATTTCGATTTCTTGGCTTTATATATCTTGTGGAAAGGACGAAACACCGGTCAAAAGACCTTTTTAATTTCTACTCTTGTAGATCAGGAAAAGACCAACCATGCATTTTTTTAAAGAATTCTCGACCTCGAGACAAATGGCAGTATTCATCCACAATTTTAAAAGAAAAGGGGGGATTGGGGGGTACAGTGCAGGGGAAAGAATAGTAGACATAATAGCAACAGACATACAAACTAAAGAATTACAAAAACAAATTACAAAAATTCAAAATTTTCGGGTTTATTACAGGGACAGCAGAGATCCACTTTGGCCGCGGCTCGAGGGGGTTGGGGTTGCGCCTTTTCCAAGGCAGCCCTGGGTTTGCGCAGGGACGCGGCTGCTCTGGGCGTGGTTCCGGGAAACGCAGCGGCGCCGACCCTGGGACTCGCACATTCTTCACGTCCGTTCGCAGCGTCACCCGGATCTTCGCCGCTACCCTTGTGGGCCCCCCGGCGACGCTTCCTGCTCCGCCCCTAAGTCGGGAAGGTTCCTTGCGGTTCGCGGCGTGCCGGACGTGACAAACGGAAGCCGCACGTCTCACTAGTACCCTCGCAGACGGACAGCGCCAGGGAGCAATGGCAGCGCGCCGACCGCGATGGGCTGTGGCCAATAGCGGCTGCTCAGCAGGGCGCGCCGAGAGCAGCGGCCGGGAAGGGGCGGTGCGGGAGGCGGGGTGTGGGGCGGTAGTGTGGGCCCTGTTCCTGCCCGCGCGGTGTTCCGCATTCTGCAAGCCTCCGGAGCGCACGTCGGCAGTCGGCTCCCTCGTTGACCGAATCACCGACCTCTCTCCCCAGGGGGATCCACCGGAGCTTACCATGACCGAGTACAAGCCCACGGTGCGCCTCGCCACCCGCGACGACGTCCCCAGGGCCGTACGCACCCTCGCCGCCGCGTTCGCCGACTACCCCGCCACGCGCCACACCGTCGATCCGGACCGCCACATCGAGCGGGTCACCGAGCTGCAAGAACTCTTCCTCACGCGCGTCGGGCTCGACATCGGCAAGGTGTGGGTCGCGGACGACGGCGCCGCGGTGGCGGTCTGGACCACGCCGGAGAGCGTCGAAGCGGGGGCGGTGTTCGCCGAGATCGGCCCGCGCATGGCCGAGTTGAGCGGTTCCCGGCTGGCCGCGCAGCAACAGATGGAAGGCCTCCTGGCGCCGCACCGGCCCAAGGAGCCCGCGTGGTTCCTGGCCACCGTCGGCGTCTCGCCCGACCACCAGGGCAAGGGTCTGGGCAGCGCCGTCGTGCTCCCCGGAGTGGAGGCGGCCGAGCGCGCCGGGGTGCCCGCCTTCCTGGAAACCTCCGCGCCCCGCAACCTCCCCTTCTACGAGCGGCTCGGCTTCACCGTCACCGCCGACGTCGAGGTGCCCGAAGGACCGCGCACCTGGTGCATGACCCGCAAGCCCGGTGCCTGA

**pCMV- enErCas12a -T2A-Egfp for gene editing in mammalian cells**

CMV promoter in red, NSL in blue, enErCas12a in orange, T2A in purple, eGFP in green

GTGATGCGGTTTTGGCAGTACATCAATGGGCGTGGATAGCGGTTTGACTCACGGGGATTTCCAAGTCTCCACCCCATTGACGTCAATGGGAGTTTGTTTTGGCACCAAAATCAACGGGACTTTCCAAAATGTCGTAACAACTCCGCCCCATTGACGCAAATGGGCGGTAGGCGTGTACGGTGGGAGGTCTATATAAGCAGAGCTGGTTTAGTGAACCGTCAGATCCGCTAGAGATCCGCGGCCGCTAATACGACTCACTATAGGGAGAGCCGCCACCATGAAACGGACAGCCGACGGAAGCGAGTTCGAGTCACCAAAGAAGAAGCGGAAAGTCAATAACGGAACTAATAACTTCCAAAACTTCATCGGGATCAGTTCCTTGCAGAAAACTCTCCGGAATGCTCTCATCCCAACTGAGACTACTCAGCAGTTCATTGTTAAGAATGGAATCATAAAAGAGGACGAGCTTAGGGGGGAAAATAGGCAAATCCTCAAGGATATCATGGATGACTATTATAGGGGCTTTATATCCGAGACACTGAGCAGCATTGATGATATAGACTGGACCTCTCTTTTCGAAAAGATGGAAATACAACTTAAAAATGGAGATAACAAGGACACCCTGATAAAGGAACAGACCGAATATAGGAAGGCAATTCATAAAAAGTTTGCTAACGATGATAGGTTTAAAAACATGTTCTCAGCAAAACTCATTTCAGATATACTGCCCGAATTCGTTATCCACAACAACAACTACTCCGCTAGCGAAAAAGAGGAAAAGACCCAAGTCATAAAGCTGTTCTCTCGATTCGCGACGAGTTTTAAAGATTATTTCCGAAATCGCGCAAACTGTTTCTCAGCTGATGATATCAGCAGCTCATCCTGTCATCGGATCGTTAACGATAATGCTGAAATCTTCTTCTCCAATGCACTTGTTTATAGGCGCATTGTTAAATCTCTCTCAAACGATGATATCAATAAGATTTCCGGCGATATGAAGGACAGTCTTAAGGAGATGAGCCTCGAAGAGATATACTCATACGAGAAATATGGCGAATTTATCACCCAGGAAGGGATTTCCTTCTATAATGACATTTGCGGCAAAGTCAATTCCTTCATGAACCTGTATTGCCAAAAAAATAAAGAAAACAAGAACCTCTATAAGCTGCAAAAGTTGCATAAGCAAATACTTTGTATCGCGGATACAAGCTATGAAGTTCCCTACAAGTTCGAGAGTGATGAGGAGGTGTATCAATCTGTCAATGGTTTCCTTGATAATATTTCTTCTAAGCATATTGTTGAACGACTCCGAAAGATAGGAGACAACTATAATGGATACAATTTGGATAAAATCTACATCGTGTCTAAATTTTACGAGAGTGTGTCACAAAAAACATATAGAGACTGGGAGACAATTAATACCGCCCTGGAGATACATTACAACAATATACTTCCCGGGAACGGGAAGTCTAAGGCAGACAAGGTGAAGAAAGCCGTGAAGAACGACTTGCAAAAGTCAATTACCGAAATCAATGAGCTTGTTTCAAACTATAAACTTTGTTCAGATGACAATATTAAAGCCGAAACCTATATTCATGAAATCTCTCATATTCTGAATAACTTTGAGGCGCAAGAACTGAAATATAACCCAGAAATACACCTCGTTGAGTCCGAACTGAAAGCAAGCGAACTGAAAAATGTTTTGGACGTGATAATGAACGCTTTTCATTGGTGCTCAGTCTTTATGACAGAGGAGCTTGTTGACAAGGATAACAATTTCTATGCGGAACTGGAAGAGATTTACGACGAAATCTATCCGGTCATATCCCTGTATAACCTGGTTCGCAACTATGTCACGCAAAAACCATACAGCACGAAGAAGATTAAACTGAACTTTGGTATTCCGACGCTGGCCCGAGGATGGTCAAAATCTAACGAATACTCAAACAATGCCATAATCCTGATGCGAAATAACCTCTACTACCTTGGAATCTTTAATGCTAAAAATAAACCCGATCGAAAAATTATCGAAGGGAACACGAGTGAAAACAAAGGTGATTATAAAAAAATGATATATAATCTGCTTCCAGGACCAAATAAGATGATACCCAAAGTTTTCCTTTCTTCAAAGACCGGCGTCGAGACATATAAACCATCCGCGTACATACTTGAAGGCTACAAACAAAATAAACATATCAAATCATCTAAGGATTTTGACATTACGTTCTGTCATGATTTGATTGACTATTTCAAAAATTGCATAGCCATTCATCCAGAGTGGAAAAACTTTGGGTTTGACTTCTCTGATACCAGTACATATGAAGACATAAGTGGATTTTACCGAGAAGTAGAGCTCCAAGGTTATAAAATAGACTGGACCTATATATCTGAAAAGGATATAGACCTTTTGCAAGAGAAGGGACAGCTTTATCTTTTCCAAATCTACAACAAAGACTTCAGTAAGAAAAGTACCGGGAATGACAATCTTCATACCATGTATCTGAAGAACCTGTTCTCCGAAGAAAATCTGAAGGACATAGTCCTGAAGCTTAATGGCGAAGCGGAAATTTTTTTCCGAAAGAGCTCTATTAAGAACCCCATAATACATAAGAAGGGAAGCATTCTCGTTAATCGAACGTATGAGGCCGAAGAGAAAGATCAATTTGGGAATATCCAAATCGTTCGAAAGAACATACCAGAAAATATTTACCAAGAATTGTACAAATATTTTAACGATAAAAGCGACAAAGAACTGTCTGATGAAGCTGCTAAGCTGAAAAACGTCGTCGGCCATCATGAGGCCGCGACGAATATAGTCAAGGATTACCGATATACATACGATAAGTATTTCCTGCATATGCCCATCACTATCAACTTTAAGGCAAATAAGACTGGATTCATTAATGACAGAATACTGCAATACATAGCTAAAGAAAAAGATTTGCATGTTATTGGCATTGACAGGGGTGAGCGCAATCTTATCTATGTAAGCGTCATTGATACTTGCGGGAATATCGTAGAGCAGAAGTCATTTAATATTGTAAATGGGTACGATTACCAAATCAAGTTGAAGCAGCAAGAGGGAGCACGACAGATTGCCCGCAAGGAGTGGAAAGAGATCGGAAAGATAAAGGAGATCAAGGAGGGGTATTTGTCCCTTGTTATACACGAAATTTCCAAGATGGTAATCAAGTACAACGCTATAATTGCTATGGAGGATCTCTCCTATGGATTTAAAAAGGGAAGATTTAAAGTCGAGCGGCAGGTATATCAGAAATTTGAAACAATGCTTATTAATAAACTTAATTATCTCGTTTTCAAAGACATTAGTATCACCGAAAACGGTGGGCTGTTGAAGGGCTATCAACTTACGTACATACCAGATAAGCTTAAGAATGTGGGTCACCAATGCGGATGCATATTCTACGTGCCCGCAGCTTATACAAGCAAAATCGACCCAACAACGGGTTTCGTAAACATATTTAAGTTCAAGGATCTCACCGTGGATGCCAAGCGAGAGTTCATAAAAAAATTTGACTCAATCAGATATGACTCAGAAAAGAATCTTTTTTGTTTTACCTTCGACTACAATAATTTCATTACACAAAATACGGTTATGAGCAAGTCATCCTGGTCCGTATATACGTATGGAGTGCGCATAAAGCGGAGATTCGTTAACGGGCGATTTTCTAATGAGTCCGATACAATCGATATAACAAAGGATATGGAAAAAACTCTGGAAATGACTGATATAAATTGGAGGGACGGTCATGACCTCAGGCAAGACATTATCGATTATGAGATCGTGCAACATATTTTTGAGATCTTTCGGTTGACTGTCCAAATGAGGAACTCTCTGTCTGAATTGGAAGATAGGGACTACGATCGCCTGATAAGCCCCGTGTTGAACGAGAATAACATATTCTACGATTCCGCGAAAGCCGGGGATGCGCTCCCTAAGGACGCCGATGCAAATGGGGCCTATTGTATTGCTTTGAAAGGGCTGTACGAAATCAAACAGATCACCGAAAACTGGAAAGAAGACGGGAAGTTTAGTCGGGATAAACTGAAGATATCCAACAAGGACTGGTTTGACTTTATCCAAAATAAGCGATATTTGAGTGGTGGCGGCTCAAAGCGTCCTGCTGCTACTAAGAAAGCTGGTCAAGCTAAGAAAAAGAAATCTAGTGAGGGCCGCGGCAGCCTGCTGACCTGCGGCGACGTGGAGGAAAACCCCGGCCCCGATTCCATGGTGAGCAAGGGCGAGGAGCTGTTCACCGGGGTGGTGCCCATCCTGGTCGAGCTGGACGGCGACGTAAACGGCCACAAGTTCAGCGTGTCCGGCGAGGGCGAGGGCGATGCCACCTACGGCAAGCTGACCCTGAAGTTCATCTGCACCACCGGCAAGCTGCCCGTGCCCTGGCCCACCCTCGTGACCACCCTGACCTACGGCGTGCAGTGCTTCAGCCGCTACCCCGACCACATGAAGCAGCACGACTTCTTCAAGTCCGCCATGCCCGAAGGCTACGTCCAGGAGCGCACCATCTTCTTCAAGGACGACGGCAACTACAAGACCCGCGCCGAGGTGAAGTTCGAGGGCGACACCCTGGTGAACCGCATCGAGCTGAAGGGCATCGACTTCAAGGAGGACGGCAACATCCTGGGGCACAAGCTGGAGTACAACTACAACAGCCACAACGTCTATATCATGGCCGACAAGCAGAAGAACGGCATCAAGGTGAACTTCAAGATCCGCCACAACATCGAGGACGGCAGCGTGCAGCTCGCCGACCACTACCAGCAGAACACCCCCATCGGCGACGGCCCCGTGCTGCTGCCCGACAACCACTACCTGAGCACCCAGTCCGCCCTGAGCAAAGACCCCAACGAGAAGCGCGATCACATGGTCCTGCTGGAGTTCGTGACCGCCGCCGGGATCACTCTCGGCATGGACGAGCTGTACAAGTAA

**enAsCa12a**

ATGACACAGTTCGAGGGCTTTACCAATCTGTACCAGGTGTCCAAGACCCTGAGGTTCGAGCTGATCCCTCAGGGCAAGACCCTGAAGCACATCCAGGAGCAGGGCTTTATCGAGGAGGACAAGGCCAGAAATGACCACTACAAGGAGCTGAAGCCCATCATCGACAGAATCTACAAGACATACGCCGACCAGTGTCTGCAGCTGGTGCAGCTGGACTGGGAGAACCTGAGCGCCGCCATCGACTCCTACAGAAAGGAGAAGACAGAGGAGACAAGGAATGCCCTGATCGAGGAGCAGGCCACCTACAGGAACGCCATCCACGACTACTTCATCGGCAGAACAGACAATCTGACAGACGCCATCAATAAGAGACACGCCGAGATCTACAAGGGCCTGTTCAAGGCCGAGCTGTTCAATGGCAAGGTGCTGAAGCAGCTGGGCACCGTGACCACAACCGAGCACGAGAATGCCCTGCTGAGGAGCTTTGATAAGTTCACCACATACTTTTCCGGCTTTTACAGGAATAGAAAGAATGTGTTTAGCGCCGAGGATATCAGCACCGCCATCCCTCACAGAATCGTGCAGGACAATTTCCCCAAGTTTAAGGAGAACTGTCACATCTTCACCAGACTGATCACAGCCGTGCCCTCCCTGAGGGAGCACTTCGAGAACGTGAAGAAGGCCATCGGCATCTTCGTGAGCACCTCCATCGAGGAGGTGTTCAGCTTCCCTTTTTACAACCAGCTGCTGACCCAGACACAGATCGACCTGTACAACCAGCTCCTGGGCGGCATCAGCAGAGAGGCCGGCACAGAGAAGATCAAGGGCCTGAACGAGGTGCTGGCCCTGGCCATCCAGAAGAATGACGAGACAGCCCACATCATCGCCTCCCTGCCTCACAGGTTCATCCCCCTGTTCAAGCAGATCCTGAGCGATAGGAATACCCTGAGCTTTATCCTGGAGGAGTTTAAGAGCGACGAGGAGGTGATCCAGTCCTTTTGTAAGTACAAGACACTGCTGAGAAATGAGAACGTGCTGGAGACAGCCGAGGCCCTGTTCAATGAGCTGAACTCCATCGATCTGACCCACATCTTCATCAGCCACAAGAAGCTGGAGACAATCAGCAGCGCCCTGTGCGACCACTGGGATACCCTGAGGAACGCCCTGTACGAGAGGAGGATCTCCGAGCTGACAGGCAAGATCACAAAGTCCGCCAAGGAGAAGGTGCAGAGAAGCCTGAAGCACGAGGACATCAATCTGCAGGAGATCATCTCCGCCGCCGGCAAGGAGCTGTCCGAGGCTTTCAAGCAGAAGACAAGCGAGATCCTGTCCCACGCCCACGCCGCCCTGGACCAACCTCTGCCTACAACCCTGAAGAAGCAGGAGGAGAAGGAGATCCTGAAGTCCCAGCTGGACTCCCTGCTGGGCCTGTACCACCTGCTGGATTGGTTCGCCGTGGACGAGTCCAACGAGGTGGACCCCGAGTTTTCCGCCAGGCTGACAGGCATCAAGCTGGAGATGGAGCCCTCCCTGTCCTTTTACAACAAGGCCAGAAACTACGCCACCAAGAAGCCTTACTCCGTGGAGAAGTTCAAGCTGAACTTTCAGATGCCCACACTGGCCAGAGGCTGGGATGTGAATAGAGAGAAGAATAATGGCGCCATCCTGTTCGTGAAGAACGGCCTGTACTACCTGGGCATCATGCCCAAGCAGAAGGGCAGATACAAGGCCCTGTCCTTCGAGCCCACCGAGAAGACCTCCGAGGGCTTCGACAAGATGTACTACGATTACTTCCCTGACGCCGCCAAGATGATCCCTAAGTGCTCCACCCAGCTGAAGGCCGTGACAGCCCACTTCCAGACACACACAACCCCCATCCTGCTGTCCAATAATTTCATCGAGCCTCTGGAGATCACAAAGGAGATCTACGATCTGAACAATCCCGAGAAGGAGCCCAAGAAGTTCCAGACCGCCTACGCCAAGAAGACAGGCGACCAGAAGGGCTACAGGGAGGCCCTGTGTAAGTGGATCGACTTCACCAGAGACTTTCTGTCCAAGTACACAAAGACCACCAGCATCGACCTGTCCTCCCTGAGGCCTAGCTCCCAGTACAAGGACCTGGGCGAGTACTACGCCGAGCTGAACCCTCTGCTGTACCACATCAGCTTTCAGAGAATCGCCGAGAAGGAGATCATGGATGCCGTGGAGACAGGCAAGCTGTACCTGTTCCAGATCTACAATAAGGACTTCGCCAAGGGCCACCACGGCAAGCCTAACCTGCACACCCTGTACTGGACCGGCCTGTTTTCCCCTGAGAACCTGGCCAAGACCTCCATCAAGCTGAATGGCCAGGCCGAGCTGTTTTACAGGCCTAAGTCCAGAATGAAGAGAATGGCCCACAGACTGGGCGAGAAGATGCTGAATAAGAAGCTGAAGGACCAGAAGACCCCTATCCCTGACACCCTGTACCAGGAGCTGTACGACTACGTGAATCACAGACTGTCCCACGATCTGTCCGATGAGGCCAGGGCCCTGCTGCCCAATGTGATCACCAAGGAGGTGTCCCACGAGATCATCAAGGACAGAAGGTTTACCAGCGACAAGTTCTTTTTTCACGTGCCCATCACCCTGAACTACCAGGCCGCCAATTCCCCCAGCAAGTTCAATCAGAGAGTGAATGCCTACCTGAAGGAGCACCCTGAGACACCTATCATCGGCATCGACAGGGGCGAGAGGAACCTGATCTACATCACAGTGATCGACAGCACCGGCAAGATCCTGGAGCAGAGAAGCCTGAATACAATCCAGCAGTTTGATTACCAGAAGAAGCTGGACAATAGGGAGAAGGAGAGGGTGGCCGCCAGACAGGCCTGGAGCGTGGTGGGAACCATCAAGGACCTGAAGCAGGGCTACCTGAGCCAGGTGATCCACGAGATCGTGGACCTGATGATCCACTACCAGGCCGTGGTGGTGCTGGAGAATCTGAACTTTGGCTTCAAGAGCAAGAGAACAGGCATCGCCGAGAAAGCCGTGTACCAGCAGTTTGAGAAGATGCTCATCGACAAGCTGAACTGTCTGGTGCTGAAGGACTACCCTGCCGAGAAGGTGGGCGGCGTGCTGAACCCTTACCAGCTGACCGATCAGTTTACATCCTTTGCCAAGATGGGCACCCAGAGCGGCTTTCTGTTCTACGTGCCCGCCCCCTACACCTCCAAGATCGACCCCCTGACCGGCTTCGTGGATCCCTTTGTGTGGAAGACCATCAAGAACCACGAGTCCAGGAAGCACTTCCTGGAGGGCTTCGATTTTCTGCACTACGATGTGAAGACCGGCGATTTTATCCTGCACTTTAAGATGAATAGGAATCTGAGCTTTCAGAGGGGCCTGCCCGGCTTCATGCCTGCCTGGGACATCGTGTTTGAGAAGAATGAGACACAGTTTGATGCCAAGGGCACCCCTTTCATCGCCGGCAAGAGAATCGTGCCCGTGATCGAGAACCACAGATTCACAGGCAGGTACAGAGACTTGTACCCCGCCAACGAGCTGATCGCCCTGCTGGAGGAGAAGGGCATCGTGTTCAGGGATGGCAGCAATATCCTGCCTAAGCTGCTGGAGAACGACGACAGCCACGCCATCGATACAATGGTGGCCCTGATCAGATCCGTGCTGCAGATGAGGAACAGCAACGCCGCCACCGGCGAGGATTACATCAACAGCCCTGTGAGGGATCTGAACGGCGTGTGCTTCGACAGCAGATTCCAGAACCCTGAGTGGCCTATGGATGCCGACGCCAATGGCGCCTACCACATCGCCCTGAAGGGCCAGCTGCTGCTGAATCACCTGAAGGAGTCCAAGGACCTGAAACTGCAGAACGGCATCAGCAACCAGGATTGGCTGGCCTACATCCAGGAGCTGAGGAAC

**impLbCas12a**

ATGAGCAAGCTGGAGAAGTTTACAAATTGCTACTCCCTGAGCAAGACCCTGAGATTCAAGGCCATCCCCGTGGGCAAGACCCAGGAGAACATCGATAATAAGAGGCTGCTGGTGGAGGACGAGAAGAGAGCCGAGGATTACAAGGGCGTGAAGAAGCTGCTGGACAGATACTACCTGTCCTTCATCAATGATGTGCTGCACTCCATCAAGCTGAAGAACCTGAACAACTACATCAGCCTGTTCAGGAAGAAGACCAGAACAGAGAAGGAGAATAAGGAGCTGGAGAATCTGGAGATCAACCTGAGAAAGGAGATCGCCAAGGCCTTCAAGGGCAACGAGGGCTACAAGTCCCTGTTTAAGAAGGACATCATCGAGACAATCCTGCCTGAGTTCCTGGACGACAAGGATGAGATCGCCCTGGTGAATTCCTTCAATGGCTTCACCACCGCCTTCACAGGCTTCTTCaggAACAGGGAGAACATGTTCAGCGAGGAGGCCAAGAGCACCAGCATCGCCTTTAGATGCATCAATGAGAATCTGACCAGATACATCAGCAACATGGATATCTTCGAGAAGGTGGACGCCATCTTCGACAAGCACGAGGTGCAGGAGATCAAGGAGAAGATCCTGAATTCCGATTACGATGTGGAGGATTTTTTCGAGGGCGAGTTTTTTAACTTCGTGCTGACACAGGAGGGCATCGACGTGTACAACGCCATCATCGGCGGCTTTGTGACAGAGTCCGGCGAGAAGATCAAGGGCCTGAATGAGTACATCAATCTGTACAACCAGAAGACCAAGCAGAAGCTGCCTAAGTTTAAGCCCCTGTACAAGCAGGTGCTGTCCGATAGGGAGAGCCTGTCCTTCTACGGCGAGGGCTACACATCCGACGAGGAGGTGCTGGAGGTGTTCAGGAACACCCTGAACAAGAACTCCGAGATCTTCAGCTCCATCAAGAAGCTGGAGAAACTGTTCAAGAATTTCGATGAGTACTCCAGCGCCGGCATCTTCGTGAAGAATGGCCCCGCCATCTCCACCATCTCCAAGGATATCTTTGGCGAGTGGAACGTGATCAGGGACAAGTGGAACGCCGAGTACGATGACATCCACCTGAAGAAGAAGGCCGTGGTGACCGAGAAGTACGAGGACGACAGGAGGAAGTCCTTTAAGAAGATCGGCAGCTTCAGCCTGGAGCAGCTGCAGGAGTACGCCGACGCCGATCTGTCCGTGGTGGAGAAGCTGAAGGAGATCATCATCCAGAAGGTGGACGAGATCTACAAGGTGTACGGCAGCAGCGAGAAGCTGTTTGACGCCGACTTTGTGCTGGAGAAGAGCCTGAAGAAGAATGACGCCGTGGTGGCCATCATGAAGGACCTGCTGGACAGCGTGAAGTCCTTTGAGAACTACATCAAGGCCTTTTTCGGCGAGGGCAAGGAGACAAACAGGGACGAGTCCTTCTACGGAGATTTCGTGCTGGCCTACGACATCCTGCTGAAGGTGGACCACATCTACGACGCCATCAGAAATTACGTGACCCAGAAGCCCTACAGCAAGGATAAGTTCAAGCTGTACTTTCAGAATCCTCAGTTTATGAGGGGCTGGGACAAGGACGTGGAGACAGATAGGAGGGCTACAATCCTGAGGTACGGCTCCAAGTACTACCTGGCCATCATGGATAAGAAGTACGCCAAGTGCCTGCAGAAGATCGACAAGGACGATGTGAATGGCAACTACGAGAAGATCAACTACAAGCTGCTGCCCGGCCCTAATAAGATGCTGCCTAGGGTGTTTTTCAGCAAGAAGTGGATGGCCTACTACAATCCCTCCGAGGATATCCAGAAGATCTACAAGAACGGCACCTTTAAGAAGGGCGATATGTTTAACCTGAACGACTGTCACAAGCTGATCGATTTCTTCAAGGACAGCATCAGCAGGTACCCCAAGTGGTCCAACGCCTACGACTTTAATTTCTCCGAGACAGAGAAGTACAAGGATATCGCCGGCTTTTACAGGGAGGTGGAGGAGCAGGGCTACAAGGTGAGCTTCGAGAGCGCCAGCAAGAAGGAGGTGGACAAGCTGGTGGAGGAGGGCAAGCTGTACATGTTTCAGATCTACAATAAGGATTTCAGCGATAAGTCCCACGGCACCCCCAATCTGCACACCATGTACTTTAAGCTGCTGTTCGACGAGAATAATCACGGCCAGATCAGACTGAGCGGCGGCGCCGAGCTGTTCATGAGAAGGGCCAGCCTGAAGAAGGAGGAGCTGGTGGTGCACCCTGCCAACAGCCCTATCGCCAACAAGAATCCCGACAACCCCAAGAAGACAACAACCCTGTCCTACGATGTGTACAAGGATAAGAGGTTTTCCGAGGACCAGTACGAGCTGCACATCCCTATCGCCATCAACAAGTGTCCTAAGAACATCTTCAAGATCAACACAGAGGTGAGAGTGCTGCTGAAGCACGATGATAACCCCTACGTGATCGGCATCgatAGAGGCGAGAGAAACCTGCTGTACATCGTGGTGGTGGATGGCAAGGGCAATATCGTGGAGCAGTACAGCCTGAACGAGATCATCAACAACTTCAACGGCATCAGGATCAAGACAGATTACCACTCCCTGCTGGACAAGAAGGAGAAGGAGAGGTTTGAGGCCAGGCAGAACTGGACCAGCATCGAGAACATCAAGGAGCTGAAGGCCGGCTACATCAGCCAGGTGGTGCACAAGATCTGTGAGCTGGTGGAGAAGTACGATGCCGTGATCGCCCTGGAGGATCTGAACAGCGGCTTCAAGAATAGCAGAGTGAAGGTGGAGAAGCAGGTGTACCAGAAGTTCGAGAAGATGCTGATCGACAAGCTGAACTACATGGTGGATAAGAAGAGCAATCCTTGTGCCACCGGCGGCGCCCTGAAGGGATACCAGATCACAAATAAGTTCGAGAGCTTTAAGTCCATGTCCACACAGAATGGCTTTATCTTTTACATCCCCGCCTGGCTGACATCCAAGATCGATCCCAGCACCGGCTTCGTGAACCTGCTGAAGACAAAGTACACAAGCATCGCCGACTCCAAGAAGTTTATCTCCAGCTTTGATAGGATCATGTACGTGCCCGAGGAGGATCTGTTTGAGTTTGCCCTGGACTACAAGAACTTCAGCAGGACCGATGCCGACTACATCAAGAAGTGGAAGCTGTACTCCTACGGCAATAGGATCAGAATCTTTAGGAACCCTAAGAAGAACAACGTGTTCGATTGGGAGGAGGTGTGTCTGACCAGCGCCTACAAGGAGCTGTTCAACAAGTACGGCATCAACTACCAGCAGGGCGATATCAGAGCCCTGCTGTGCGAGCAGAGCGACAAGGCCTTCTACAGCTCCTTTATGGCCCTGATGTCCCTGATGCTGCAGATGAGGAATAGCATCACAGGCAGAACAGACGTGGATTTTCTGATCTCCCCCGTGAAGAATAGCGACGGCATCTTTTACGACTCCAGGAACTACGAGGCCCAGGAGAACGCCATCCTGCCTAAGAATGCCGACGCCAATGGCGCCTACAACATCGCCAGAAAGGTGCTGTGGGCCATCGGCCAGTTTAAGAAGGCCGAGGATGAGAAGCTGGACAAGGTGAAGATCGCCATCAGCAATAAGGAGTGGCTGGAGTACGCCCAGACAAGCGTGAAGCAC

**pCMV-TAD8-enErCas12a-GFP**

CMV promoter in red, NSL in blue, TAD8 in gray, denErCas12a in orange, T2A in purple, eGFP in green

GACATTGATTATTGACTAGTTATTAATAGTAATCAATTACGGGGTCATTAGTTCATAGCCCATATATGGAGTTCCGCGTTACATAACTTACGGTAAATGGCCCGCCTGGCTGACCGCCCAACGACCCCCGCCCATTGACGTCAATAATGACGTATGTTCCCATAGTAACGCCAATAGGGACTTTCCATTGACGTCAATGGGTGGAGTATTTACGGTAAACTGCCCACTTGGCAGTACATCAAGTGTATCATATGCCAAGTACGCCCCCTATTGACGTCAATGACGGTAAATGGCCCGCCTGGCATTATGCCCAGTACATGACCTTATGGGACTTTCCTACTTGGCAGTACATCTACGTATTAGTCATCGCTATTACCATGGTGATGCGGTTTTGGCAGTACATCAATGGGCGTGGATAGCGGTTTGACTCACGGGGATTTCCAAGTCTCCACCCCATTGACGTCAATGGGAGTTTGTTTTGGCACCAAAATCAACGGGACTTTCCAAAATGTCGTAACAACTCCGCCCCATTGACGCAAATGGGCGGTAGGCGTGTACGGTGGGAGGTCTATATAAGCAGAGCTGGTTTAGTGAACCGTCAGATCCGCTAGAGATCCGCGGCCGCTAATACGACTCACTATAGGGAGAGCCGCCACCATGAAACGGACAGCCGACGGAAGCGAGTTCGAGTCACCAAAGAAGAAGCGGAAAGTCAGCGAGGTGGAGTTCAGCCACGAGTACTGGATGAGACACGCCCTGACACTGGCCAAGAGGGCCAGAGACGAGAGAGAGGTGCCTGTGGGCGCCGTGCTGGTGCTGAACAACAGAGTGATCGGCGAGGGCTGGAACAGAGCCATCGGCCTGCACGACCCTACAGCCCACGCCGAGATCATGGCCCTGAGGCAGGGCGGCCTGGTCATGCAGAATTACAGACTGATCGATGCCACCCTGTACGTGACCTTTGAGCCTTGTGTGATGTGCGCCGGCGCCATGATCCACTCCAGAATCGGCAGAGTGGTGTTTGGCGTGAGAAACTCCAAGAGGGGCGCCGCCGGCAGCCTGATGAATGTGCTGAACTACCCTGGCATGAACCACAGGGTGGAGATCACAGAGGGCATCCTGGCCGATGAGTGCGCCGCCCTGCTGTGCGACTTCTACAGGATGCCCAGGCAGGTGTTCAATGCCCAGAAGAAGGCCCAGAGCAGCATCAATTCTGGAGGATCTAGCGGAGGATCCTCTGGCAGCGAGACACCAGGAACAAGCGAGTCAGCAACACCAGAGAGCAGTGGCGGCAGCAGCGGCGGCAGCAATAACGGAACTAATAACTTCCAAAACTTCATCGGGATCAGTTCCTTGCAGAAAACTCTCCGGAATGCTCTCATCCCAACTGAGACTACTCAGCAGTTCATTGTTAAGAATGGAATCATAAAAGAGGACGAGCTTAGGGGGGAAAATAGGCAAATCCTCAAGGATATCATGGATGACTATTATAGGGGCTTTATATCCGAGACACTGAGCAGCATTGATGATATAGACTGGACCTCTCTTTTCGAAAAGATGGAAATACAACTTAAAAATGGAGATAACAAGGACACCCTGATAAAGGAACAGACCGAATATAGGAAGGCAATTCATAAAAAGTTTGCTAACGATGATAGGTTTAAAAACATGTTCTCAGCAAAACTCATTTCAGATATACTGCCCGAATTCGTTATCCACAACAACAACTACTCCGCTAGCGAAAAAGAGGAAAAGACCCAAGTCATAAAGCTGTTCTCTCGATTCGCGACGAGTTTTAAAGATTATTTCCGAAATCGCGCAAACTGTTTCTCAGCTGATGATATCAGCAGCTCATCCTGTCATCGGATCGTTAACGATAATGCTGAAATCTTCTTCTCCAATGCACTTGTTTATAGGCGCATTGTTAAATCTCTCTCAAACGATGATATCAATAAGATTTCCGGCGATATGAAGGACAGTCTTAAGGAGATGAGCCTCGAAGAGATATACTCATACGAGAAATATGGCGAATTTATCACCCAGGAAGGGATTTCCTTCTATAATGACATTTGCGGCAAAGTCAATTCCTTCATGAACCTGTATTGCCAAAAAAATAAAGAAAACAAGAACCTCTATAAGCTGCAAAAGTTGCATAAGCAAATACTTTGTATCGCGGATACAAGCTATGAAGTTCCCTACAAGTTCGAGAGTGATGAGGAGGTGTATCAATCTGTCAATGGTTTCCTTGATAATATTTCTTCTAAGCATATTGTTGAACGACTCCGAAAGATAGGAGACAACTATAATGGATACAATTTGGATAAAATCTACATCGTGTCTAAATTTTACGAGAGTGTGTCACAAAAAACATATAGAGACTGGGAGACAATTAATACCGCCCTGGAGATACATTACAACAATATACTTCCCGGGAACGGGAAGTCTAAGGCAGACAAGGTGAAGAAAGCCGTGAAGAACGACTTGCAAAAGTCAATTACCGAAATCAATGAGCTTGTTTCAAACTATAAACTTTGTTCAGATGACAATATTAAAGCCGAAACCTATATTCATGAAATCTCTCATATTCTGAATAACTTTGAGGCGCAAGAACTGAAATATAACCCAGAAATACACCTCGTTGAGTCCGAACTGAAAGCAAGCGAACTGAAAAATGTTTTGGACGTGATAATGAACGCTTTTCATTGGTGCTCAGTCTTTATGACAGAGGAGCTTGTTGACAAGGATAACAATTTCTATGCGGAACTGGAAGAGATTTACGACGAAATCTATCCGGTCATATCCCTGTATAACCTGGTTCGCAACTATGTCACGCAAAAACCATACAGCACGAAGAAGATTAAACTGAACTTTGGTATTCCGACGCTGGCCCGAGGATGGTCAAAATCTAACGAATACTCAAACAATGCCATAATCCTGATGCGAAATAACCTCTACTACCTTGGAATCTTTAATGCTAAAAATAAACCCGATCGAAAAATTATCGAAGGGAACACGAGTGAAAACAAAGGTGATTATAAAAAAATGATATATAATCTGCTTCCAGGACCAAATAAGATGATACCCAAAGTTTTCCTTTCTTCAAAGACCGGCGTCGAGACATATAAACCATCCGCGTACATACTTGAAGGCTACAAACAAAATAAACATATCAAATCATCTAAGGATTTTGACATTACGTTCTGTCATGATTTGATTGACTATTTCAAAAATTGCATAGCCATTCATCCAGAGTGGAAAAACTTTGGGTTTGACTTCTCTGATACCAGTACATATGAAGACATAAGTGGATTTTACCGAGAAGTAGAGCTCCAAGGTTATAAAATAGACTGGACCTATATATCTGAAAAGGATATAGACCTTTTGCAAGAGAAGGGACAGCTTTATCTTTTCCAAATCTACAACAAAGACTTCAGTAAGAAAAGTACCGGGAATGACAATCTTCATACCATGTATCTGAAGAACCTGTTCTCCGAAGAAAATCTGAAGGACATAGTCCTGAAGCTTAATGGCGAAGCGGAAATTTTTTTCCGAAAGAGCTCTATTAAGAACCCCATAATACATAAGAAGGGAAGCATTCTCGTTAATCGAACGTATGAGGCCGAAGAGAAAGATCAATTTGGGAATATCCAAATCGTTCGAAAGAACATACCAGAAAATATTTACCAAGAATTGTACAAATATTTTAACGATAAAAGCGACAAAGAACTGTCTGATGAAGCTGCTAAGCTGAAAAACGTCGTCGGCCATCATGAGGCCGCGACGAATATAGTCAAGGATTACCGATATACATACGATAAGTATTTCCTGCATATGCCCATCACTATCAACTTTAAGGCAAATAAGACTGGATTCATTAATGACAGAATACTGCAATACATAGCTAAAGAAAAAGATTTGCATGTTATTGGCATTGCCAGGGGTGAGCGCAATCTTATCTATGTAAGCGTCATTGATACTTGCGGGAATATCGTAGAGCAGAAGTCATTTAATATTGTAAATGGGTACGATTACCAAATCAAGTTGAAGCAGCAAGAGGGAGCACGACAGATTGCCCGCAAGGAGTGGAAAGAGATCGGAAAGATAAAGGAGATCAAGGAGGGGTATTTGTCCCTTGTTATACACGAAATTTCCAAGATGGTAATCAAGTACAACGCTATAATTGCTATGGAGGATCTCTCCTATGGATTTAAAAAGGGAAGATTTAAAGTCGAGCGGCAGGTATATCAGAAATTTGAAACAATGCTTATTAATAAACTTAATTATCTCGTTTTCAAAGACATTAGTATCACCGAAAACGGTGGGCTGTTGAAGGGCTATCAACTTACGTACATACCAGATAAGCTTAAGAATGTGGGTCACCAATGCGGATGCATATTCTACGTGCCCGCAGCTTATACAAGCAAAATCGACCCAACAACGGGTTTCGTAAACATATTTAAGTTCAAGGATCTCACCGTGGATGCCAAGCGAGAGTTCATAAAAAAATTTGACTCAATCAGATATGACTCAGAAAAGAATCTTTTTTGTTTTACCTTCGACTACAATAATTTCATTACACAAAATACGGTTATGAGCAAGTCATCCTGGTCCGTATATACGTATGGAGTGCGCATAAAGCGGAGATTCGTTAACGGGCGATTTTCTAATGAGTCCGATACAATCGATATAACAAAGGATATGGAAAAAACTCTGGAAATGACTGATATAAATTGGAGGGACGGTCATGACCTCAGGCAAGACATTATCGATTATGAGATCGTGCAACATATTTTTGAGATCTTTCGGTTGACTGTCCAAATGAGGAACTCTCTGTCTGAATTGGAAGATAGGGACTACGATCGCCTGATAAGCCCCGTGTTGAACGAGAATAACATATTCTACGATTCCGCGAAAGCCGGGGATGCGCTCCCTAAGGACGCCGATGCAAATGGGGCCTATTGTATTGCTTTGAAAGGGCTGTACGAAATCAAACAGATCACCGAAAACTGGAAAGAAGACGGGAAGTTTAGTCGGGATAAACTGAAGATATCCAACAAGGACTGGTTTGACTTTATCCAAAATAAGCGATATTTGTCTGGCGGCTCAAAAAGAACCGCCGACGGCAGCGAATTCGAGCCCAAGAAGAAGAGGAAAGTCGGAAGCGGAGCTACTAACTTCAGCCTGCTGAAGCAGGCTGGAGACGTGGAGGAGAACCCTGGACCTATGGTGAGCAAGGGCGAGGAGCTGTTCACCGGGGTGGTGCCCATCCTGGTCGAGCTGGACGGCGACGTAAACGGCCACAAGTTCAGCGTGTCCGGCGAGGGCGAGGGCGATGCCACCTACGGCAAGCTGACCCTGAAGTTCATCTGCACCACCGGCAAGCTGCCCGTGCCCTGGCCCACCCTCGTGACCACCCTGACCTATGGAGTGCAGTGCTTCAGCCGCTACCCCGACCACATGAAGCAGCACGACTTCTTCAAGTCCGCCATGCCCGAAGGCTACGTCCAGGAGCGCACCATCTTCTTCAAGGACGACGGCAACTACAAGACCCGCGCCGAGGTGAAGTTCGAGGGCGACACCCTGGTGAACCGCATCGAGCTGAAGGGCATCGACTTCAAGGAGGACGGCAACATCCTGGGGCACAAGCTGGAGTACAACTACAACAGCCACAACGTCTATATCATGGCCGACAAGCAGAAGAACGGCATCAAGGTGAACTTCAAGATCCGCCACAACATCGAGGACGGCAGCGTGCAGCTCGCCGACCACTACCAGCAGAACACCCCCATCGGCGACGGCCCCGTGCTGCTGCCCGACAACCACTACCTGAGCACCCAGTCCGCCCTGAGCAAAGACCCCAACGAGAAGCGCGATCACATGGTCCTGCTGGAGTTCGTGACCGCCGCCGGGATCACTCTCGGCATGGACGAGCTGTACAAGTCTGGTGGTTCTCCCAAGAAGAAAAGGAAAGTCTAA

**pHS-crRNA-****enErCas12a**

U6 promoter in gray, crRNA in brown with repeat unlined, HEf1a promoter in red, NSL in blue, enErCas12a in orange, T2A in purple, eGFP in green

GGCAGGAAGAGGGCCTATTTCCCATGATTCCTTCATATTTGCATATACGATACAAGGCTGTTAGAGAGATAATTAGAATTAATTTGACTGTAAACACAAAGATATTAGTACAAAATACGTGACGTAGAAAGTAATAATTTCTTGGGTAGTTTGCAGTTTTAAAATTATGTTTTAAAATGGACTATCATATGCTTACCGTAACTTGAAAGTATTTCGATTTCTTGGCTTTATATATCTTGTGGAAAGGACGAAACACCGGTCAAAAGACCTTTTTAATTTCTACTCTTGTAGATCGGGGTCCCCTTCTTCAAGCATTTTTTTAAGCTTGGCTCCGGTGCCCGTCAGTGGGCAGAGCGCACATCGCCCACAGTCCCCGAGAAGTTGTGGGGAGGGGTCGGCAATTGAACCGGTGCCTAGAGAAGGTGGCGCGGGGTAAACTGGGAAAGTGATGTCGTGTACTGGCTCCGCCTTTTTCCCGAGGGTGGGGGAGAACCGTATATAAGTGCAGTAGTCGCCGTGAACGTTCTTTTTCGCAACGGGTTTGCCGCCAGAACACAGGTAAGTGCCGTGTGTGGTTCCCGCGGGCCTGGCCTCTTTACGGGTTATGGCCCTTGCGTGCCTTGAATTACTTCCACCTGGCTGCAGTACGTGATTCTTGATCCCGAGCTTCGGGTTGGAAGTGGGTGGGAGAGTTCGAGGCCTTGCGCTTAAGGAGCCCCTTCGCCTCGTGCTTGAGTTGAGGCCTGGCCTGGGCGCTGGGGCCGCCGCGTGCGAATCTGGTGGCACCTTCGCGCCTGTCTCGCTGCTTTCGATAAGTCTCTAGCCATTTAAAATTTTTGATGACCTGCTGCGACGCTTTTTTTCTGGCAAGATAGTCTTGTAAATGCGGGCCAAGATCTGCACACTGGTATTTCGGTTTTTGGGGCCGCGGGCGGCGACGGGGCCCGTGCGTCCCAGCGCACATGTTCGGCGAGGCGGGGCCTGCGAGCGCGGCCACCGAGAATCGGACGGGGGTAGTCTCAAGCTGGCCGGCCTGCTCTGGTGCCTGGCCTCGCGCCGCCGTGTATCGCCCCGCCCTGGGCGGCAAGGCTGGCCCGGTCGGCACCAGTTGCGTGAGCGGAAAGATGGCCGCTTCCCGGCCCTGCTGCAGGGAGCTCAAAATGGAGGACGCGGCGCTCGGGAGAGCGGGCGGGTGAGTCACCCACACAAAGGAAAAGGGCCTTTCCGTCCTCAGCCGTCGCTTCATGTGACTCCACGGAGTACCGGGCGCCGTCCAGGCACCTCGATTAGTTCTCGAGCTTTTGGAGTACGTCGTCTTTAGGTTGGGGGGAGGGGTTTTATGCGATGGAGTTTCCCCACACTGAGTGGGTGGAGACTGAAGTTAGGCCAGCTTGGCACTTGATGTAATTCTCCTTGGAATTTGCCCTTTTTGAGTTTGGATCTTGGTTCATTCTCAAGCCTCAGACAGTGGTTCAAAGTTTTTTTCTTCCATTTCAGGTCCCGGGTAACTGATCATAATTCGACCCAAGTTTGTACAAAAAAGCAGGCTGATTACCGGAGAATTCCAATTGGCGGCCGCACCGGTGCCACCATGCCAAAGAAGAAGCGGAAGGTCGGTGGCGGCTCACCCGGGATGAATAACGGAACTAATAACTTCCAAAACTTCATCGGGATCAGTTCCTTGCAGAAAACTCTCCGGAATGCTCTCATCCCAACTGAGACTACTCAGCAGTTCATTGTTAAGAATGGAATCATAAAAGAGGACGAGCTTAGGGGGGAAAATAGGCAAATCCTCAAGGATATCATGGATGACTATTATAGGGGCTTTATATCCGAGACACTGAGCAGCATTGATGATATAGACTGGACCTCTCTTTTCGAAAAGATGGAAATACAACTTAAAAATGGAGATAACAAGGACACCCTGATAAAGGAACAGACCGAATATAGGAAGGCAATTCATAAAAAGTTTGCTAACGATGATAGGTTTAAAAACATGTTCTCAGCAAAACTCATTTCAGATATACTGCCCGAATTCGTTATCCACAACAACAACTACTCCGCTAGCGAAAAAGAGGAAAAGACCCAAGTCATAAAGCTGTTCTCTCGATTCGCGACGAGTTTTAAAGATTATTTCCGAAATCGCGCAAACTGTTTCTCAGCTGATGATATCAGCAGCTCATCCTGTCATCGGATCGTTAACGATAATGCTGAAATCTTCTTCTCCAATGCACTTGTTTATAGGCGCATTGTTAAATCTCTCTCAAACGATGATATCAATAAGATTTCCGGCGATATGAAGGACAGTCTTAAGGAGATGAGCCTCGAAGAGATATACTCATACGAGAAATATGGCGAATTTATCACCCAGGAAGGGATTTCCTTCTATAATGACATTTGCGGCAAAGTCAATTCCTTCATGAACCTGTATTGCCAAAAAAATAAAGAAAACAAGAACCTCTATAAGCTGCAAAAGTTGCATAAGCAAATACTTTGTATCGCGGATACAAGCTATGAAGTTCCCTACAAGTTCGAGAGTGATGAGGAGGTGTATCAATCTGTCAATGGTTTCCTTGATAATATTTCTTCTAAGCATATTGTTGAACGACTCCGAAAGATAGGAGACAACTATAATGGATACAATTTGGATAAAATCTACATCGTGTCTAAATTTTACGAGAGTGTGTCACAAAAAACATATAGAGACTGGGAGACAATTAATACCGCCCTGGAGATACATTACAACAATATACTTCCCGGGAACGGGAAGTCTAAGGCAGACAAGGTGAAGAAAGCCGTGAAGAACGACTTGCAAAAGTCAATTACCGAAATCAATGAGCTTGTTTCAAACTATAAACTTTGTTCAGATGACAATATTAAAGCCGAAACCTATATTCATGAAATCTCTCATATTCTGAATAACTTTGAGGCGCAAGAACTGAAATATAACCCAGAAATACACCTCGTTGAGTCCGAACTGAAAGCAAGCGAACTGAAAAATGTTTTGGACGTGATAATGAACGCTTTTCATTGGTGCTCAGTCTTTATGACAGAGGAGCTTGTTGACAAGGATAACAATTTCTATGCGGAACTGGAAGAGATTTACGACGAAATCTATCCGGTCATATCCCTGTATAACCTGGTTCGCAACTATGTCACGCAAAAACCATACAGCACGAAGAAGATTAAACTGAACTTTGGTATTCCGACGCTGGCCCGAGGATGGTCAAAATCTAACGAATACTCAAACAATGCCATAATCCTGATGCGAAATAACCTCTACTACCTTGGAATCTTTAATGCTAAAAATAAACCCGATCGAAAAATTATCGAAGGGAACACGAGTGAAAACAAAGGTGATTATAAAAAAATGATATATAATCTGCTTCCAGGACCAAATAAGATGATACCCAAAGTTTTCCTTTCTTCAAAGACCGGCGTCGAGACATATAAACCATCCGCGTACATACTTGAAGGCTACAAACAAAATAAACATATCAAATCATCTAAGGATTTTGACATTACGTTCTGTCATGATTTGATTGACTATTTCAAAAATTGCATAGCCATTCATCCAGAGTGGAAAAACTTTGGGTTTGACTTCTCTGATACCAGTACATATGAAGACATAAGTGGATTTTACCGAGAAGTAGAGCTCCAAGGTTATAAAATAGACTGGACCTATATATCTGAAAAGGATATAGACCTTTTGCAAGAGAAGGGACAGCTTTATCTTTTCCAAATCTACAACAAAGACTTCAGTAAGAAAAGTACCGGGAATGACAATCTTCATACCATGTATCTGAAGAACCTGTTCTCCGAAGAAAATCTGAAGGACATAGTCCTGAAGCTTAATGGCGAAGCGGAAATTTTTTTCCGAAAGAGCTCTATTAAGAACCCCATAATACATAAGAAGGGAAGCATTCTCGTTAATCGAACGTATGAGGCCGAAGAGAAAGATCAATTTGGGAATATCCAAATCGTTCGAAAGAACATACCAGAAAATATTTACCAAGAATTGTACAAATATTTTAACGATAAAAGCGACAAAGAACTGTCTGATGAAGCTGCTAAGCTGAAAAACGTCGTCGGCCATCATGAGGCCGCGACGAATATAGTCAAGGATTACCGATATACATACGATAAGTATTTCCTGCATATGCCCATCACTATCAACTTTAAGGCAAATAAGACTGGATTCATTAATGACAGAATACTGCAATACATAGCTAAAGAAAAAGATTTGCATGTTATTGGCATTGACAGGGGTGAGCGCAATCTTATCTATGTAAGCGTCATTGATACTTGCGGGAATATCGTAGAGCAGAAGTCATTTAATATTGTAAATGGGTACGATTACCAAATCAAGTTGAAGCAGCAAGAGGGAGCACGACAGATTGCCCGCAAGGAGTGGAAAGAGATCGGAAAGATAAAGGAGATCAAGGAGGGGTATTTGTCCCTTGTTATACACGAAATTTCCAAGATGGTAATCAAGTACAACGCTATAATTGCTATGGAGGATCTCTCCTATGGATTTAAAAAGGGAAGATTTAAAGTCGAGCGGCAGGTATATCAGAAATTTGAAACAATGCTTATTAATAAACTTAATTATCTCGTTTTCAAAGACATTAGTATCACCGAAAACGGTGGGCTGTTGAAGGGCTATCAACTTACGTACATACCAGATAAGCTTAAGAATGTGGGTCACCAATGCGGATGCATATTCTACGTGCCCGCAGCTTATACAAGCAAAATCGACCCAACAACGGGTTTCGTAAACATATTTAAGTTCAAGGATCTCACCGTGGATGCCAAGCGAGAGTTCATAAAAAAATTTGACTCAATCAGATATGACTCAGAAAAGAATCTTTTTTGTTTTACCTTCGACTACAATAATTTCATTACACAAAATACGGTTATGAGCAAGTCATCCTGGTCCGTATATACGTATGGAGTGCGCATAAAGCGGAGATTCGTTAACGGGCGATTTTCTAATGAGTCCGATACAATCGATATAACAAAGGATATGGAAAAAACTCTGGAAATGACTGATATAAATTGGAGGGACGGTCATGACCTCAGGCAAGACATTATCGATTATGAGATCGTGCAACATATTTTTGAGATCTTTCGGTTGACTGTCCAAATGAGGAACTCTCTGTCTGAATTGGAAGATAGGGACTACGATCGCCTGATAAGCCCCGTGTTGAACGAGAATAACATATTCTACGATTCCGCGAAAGCCGGGGATGCGCTCCCTAAGGACGCCGATGCAAATGGGGCCTATTGTATTGCTTTGAAAGGGCTGTACGAAATCAAACAGATCACCGAAAACTGGAAAGAAGACGGGAAGTTTAGTCGGGATAAACTGAAGATATCCAACAAGGACTGGTTTGACTTTATCCAAAATAAGCGATATTTGACTAGTGGTGGCGGCTCAAAGCGTCCTGCTGCTACTAAGAAAGCTGGTCAAGCTAAGAAAAAGAAATCTAGTGAGGGCCGCGGCAGCCTGCTGACCTGCGGCGACGTGGAGGAAAACCCCGGCCCCGATTCCATGGTGAGCAAGGGCGAGGAGCTGTTCACCGGGGTGGTGCCCATCCTGGTCGAGCTGGACGGCGACGTAAACGGCCACAAGTTCAGCGTGTCCGGCGAGGGCGAGGGCGATGCCACCTACGGCAAGCTGACCCTGAAGTTCATCTGCACCACCGGCAAGCTGCCCGTGCCCTGGCCCACCCTCGTGACCACCCTGACCTACGGCGTGCAGTGCTTCAGCCGCTACCCCGACCACATGAAGCAGCACGACTTCTTCAAGTCCGCCATGCCCGAAGGCTACGTCCAGGAGCGCACCATCTTCTTCAAGGACGACGGCAACTACAAGACCCGCGCCGAGGTGAAGTTCGAGGGCGACACCCTGGTGAACCGCATCGAGCTGAAGGGCATCGACTTCAAGGAGGACGGCAACATCCTGGGGCACAAGCTGGAGTACAACTACAACAGCCACAACGTCTATATCATGGCCGACAAGCAGAAGAACGGCATCAAGGTGAACTTCAAGATCCGCCACAACATCGAGGACGGCAGCGTGCAGCTCGCCGACCACTACCAGCAGAACACCCCCATCGGCGACGGCCCCGTGCTGCTGCCCGACAACCACTACCTGAGCACCCAGTCCGCCCTGAGCAAAGACCCCAACGAGAAGCGCGATCACATGGTCCTGCTGGAGTTCGTGACCGCCGCCGGGATCACTCTCGGCATGGACGAGCTGTACAAGGAGGGCCGCGGCAGCCTGCTGACCTGCGGCGACGTGGAGGAAAACCCCGGCCCCATGACCGAGTACAAGCCCACGGTGCGCCTCGCCACCCGCGACGACGTCCCCCGGGCCGTACGCACCCTCGCCGCCGCGTTCGCCGACTACCCCGCCACGCGCCACACCGTCGACCCGGACCGCCACATCGAGCGGGTCACCGAGCTGCAAGAACTCTTCCTCACGCGCGTCGGGCTCGACATCGGCAAGGTGTGGGTCGCGGACGACGGCGCCGCGGTGGCGGTCTGGACCACGCCGGAGAGCGTCGAAGCGGGGGCGGTGTTCGCCGAGATCGGCCCGCGCATGGCCGAGTTGAGCGGTTCCCGGCTGGCCGCGCAGCAACAGATGGAAGGCCTCCTGGCGCCGCACCGGCCCAAGGAGCCCGCGTGGTTCCTGGCCACCGTCGGCGTGTCGCCCGACCACCAGGGCAAGGGTCTGGGCAGCGCCGTCGTGCTCCCCGGAGTGGAGGCGGCCGAGCGCGCCGGGGTGCCCGCCTTCCTGGAAACCTCCGCGCCCCGCAACCTCCCCTTCTACGAGCGGCTCGGCTTCACCGTCACCGCCGACGTCGAGGTGCCCGAAGGACCGCGCACCTGGTGCATGACCCGCAAGCCCGGTGCCTGA
